# Structural Design and Challenges of Micron‐Scale Silicon‐Based Lithium‐ion Batteries

**DOI:** 10.1002/advs.202407540

**Published:** 2025-01-09

**Authors:** Wenjie He, Wei Xu, Zhigang Li, Zhaotong Hu, Jia Yang, Gang Qin, Weiming Teng, Tengfei Zhang, Wei Zhang, Zhengming Sun, Xuebin Yu

**Affiliations:** ^1^ School of Materials Science and Engineering Henan Polytechnic University Jiaozuo 454003 China; ^2^ College of Materials Science and Technology Nanjing University of Aeronautics and Astronautics Nanjing 210016 China; ^3^ Department of Materials Science Fudan University Shanghai 200433 China; ^4^ Zhejiang Baima Lake Lab Co Ltd Hangzhou 310051 China; ^5^ Jiangsu Key Laboratory of Advanced Metallic Materials School of Materials Science and Engineering Southeast University Nanjing 211189 China

**Keywords:** industrial‐scale applications, Lithium‐ion batteries, silicon‐based anodes, solid‐electrolyte interphases, structural designs

## Abstract

Currently, lithium‐ion batteries (LIBs) are at the forefront of energy storage technologies. Silicon‐based anodes, with their high capacity and low cost, present a promising alternative to traditional graphite anodes in LIBs, offering the potential for substantial improvements in energy density. However, the significant volumetric changes that silicon‐based anodes undergo during charge and discharge cycles can lead to structural degradation. Furthermore, the formation of excessive solid‐electrolyte interphases (SEIs) during cycling impedes the efficient migration of ions and electrons. This comprehensive review focuses on the structural design and optimization of micron‐scale silicon‐based anodes from both materials and systems perspectives. Significant progress is made in the development of advanced electrolytes, binders, and conductive additives that complement micron‐scale silicon‐based anodes in both half and full‐cells. Moreover, advancements in system‐level technologies, such as pre‐lithiation techniques to mitigate irreversible Li^+^ loss, have enhanced the energy density and lifespan of micron‐scale silicon‐based full cells. This review concludes with a detailed classification of the underlying mechanisms, providing a comprehensive summary to guide the development of high‐energy‐density devices. It also offers strategic insights to address the challenges associated with the large‐scale deployment of silicon‐based LIBs.

## Graphical abstract

The significant volume change of the silicon‐based anode during cycling can lead to its structural degradation and the formation of excess solid‐electrolyte interphases. For the above problems, this review focuses on the structural design and optimization of micron‐sized silicon‐based anodes from the perspectives of materials and systems, providing a comprehensive summary to guide the development of high‐energy‐density devices.

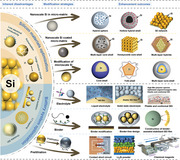

## 1 Introduction

The rapid acceleration of energy consumption, driven by the fast‐paced development of modern society, has led to a range of environmental challenges. In the pursuit of sustainable development and carbon neutrality, there is an urgent need for highly efficient and clean energy conversion solutions.^[^ ^1^, ^2^ ^]^ Today, rechargeable lithium‐ion batteries (LIBs) are widely used in the energy storage market, with applications spanning portable electronics, electric vehicles, electric aircraft, and smart grids.^[^ ^3^, ^4^, ^5^ ^]^ However, the commercial advancement of LIBs has faced significant challenges due to the diverse and evolving needs of consumers.^[^ ^6^, ^7^, ^8^ ^]^ The demands for LIBs go beyond long lifespan and environmental friendliness; there is also an increasing need for higher energy storage capacity within constrained spaces. The demands for LIBs extend beyond just long lifespan and environmental friendliness, which is also a growing need for more energy storage within limited space.^[^ ^9^, ^10^, ^11^ ^]^ As is well known, the theoretical energy density of LIBs is determined by the average operating voltage and theoretical specific capacity. As a result, silicon‐based materials (SBMs) have been explored as alternatives to commercial graphite due to their exceptionally high theoretical specific capacity (3800 mAh g^−1^), low operating voltage (<0.4 V vs Li/Li^+^), and abundant natural reserves.^[^ ^12^, ^13^, ^14^ ^]^

**Figure 1:**
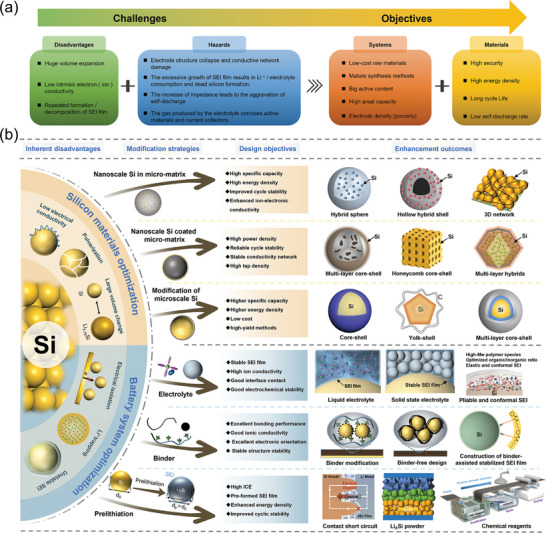
a) Challenges of Si anodes and key objectives. b) Micron‐scale silicon‐based LIBs: materials and systems perspectives.

Regrettably, the significant volumetric expansion of Si (≈300% at room temperature) induced by the formation of Li_15_Si_4_ during alloying results in considerable capacity fading (**Figure** **1**a).^[^ ^15^, ^16^ ^]^ The substantial volume change induces concentrated stress within the particles, leading to fracture and electrical isolation, which in turn causes significant capacity loss. Additionally, the low intrinsic electrical conductivity (≈10^−3^ S cm^−1^) and slow lithium‐ion diffusion rate (10^−14^–10^−13^ cm^2^ s^−1^) contribute to sluggish electrochemical kinetics.^[^ ^17^, ^18^ ^]^ Currently, a variety of strategies have been proposed to enhance the structural stability and electron/ion conductivity of SBMs.^[^ ^5^, ^12^, ^17^ ^]^ Particle size plays a crucial role in the structural and compositional evolution, electrochemical performance, mechanochemical behavior, and surface chemistry of Si anodes.^[^ ^19^ ^]^ Both nano‐Si and micro‐Si undergo a similar phase transition from crystalline to amorphous. However, the failure of micro‐Si is primarily attributed to particle pulverization and electrode cracking induced by volumetric expansion. In 2012, a critical particle size of ≈150 nm was established as a benchmark for Si anodes in LIBs with liquid electrolytes. The nanocrystallization of Si particles (by reducing their size to <150 nm) helps alleviate mechanical stress concentration during Li^+^ intercalation and deintercalation, reducing the risk of electrode degradation and fracture, and improving electrochemical stability.^[^ ^20^, ^21^, ^22^ ^]^ This critical size represents the maximum particle size that remains intact during charge and discharge cycles. Recently, the size effect of Si anodes has been shown to be relevant to all‐solid‐state batteries (ASSBs), and the application of longitudinal stacking pressure can enhance electrochemical performance within a certain range.^[^ ^23^ ^]^ The critical size of Si particles depends on the applied stacking pressure; specifically, higher stacking pressure allows for a larger critical particle size. Under stacking pressure, the critical size shifts from the nanoscale range observed in LIBs with liquid electrolytes to the microscale range characteristic of ASSBs. The above findings provide valuable theoretical insights for modifying Si anodes. However, maintaining a stable and high stacking pressure to ensure the cycling stability of Si anodes in practical applications remains a significant challenge.

However, nanocrystallization significantly increases the surface area‐to‐volume ratio of Si, and the excessive formation of solid‐electrolyte interphases (SEIs) during cycling can lead to substantial consumption of liquid electrolytes.^[^ ^24^, ^25^, ^26^ ^]^ The pores formed during Li^+^ deintercalation expose new surfaces within the silicon nanoparticles (NPs), leading to further reactions with the electrolyte and the formation of the SEI film.^[^ ^27^, ^28^ ^]^ With the passage of time, the non‐conductive species generated in the SEI film obstruct electronic conductivity, resulting in the formation of electrochemically inert silicon. This is a primary cause of the rapid capacity decay observed in nano‐silicon. In full cells, the continuous decomposition of the limited liquid electrolyte is particularly problematic and deemed unacceptable in industry.^[^ ^29^ ^]^ Furthermore, the formation of irreversible by‐products and the trapping of Li⁺ contribute to the reduction of initial Coulombic efficiency (ICE) and energy density in silicon‐based batteries. Additionally, the uncontrolled aggregation of Si NPs can lead to local stress concentrations within thick electrodes, further degrading electrochemical performance. To meet industrial standards, mass loadings must fulfill key criteria, including areal capacity loading (>3.0 mA h cm^−2^), pressing density (>1.6 g cm^−3^), and volume variation (<70%, preferably <30%).^[^ ^30^, ^31^, ^32^ ^]^ Therefore, achieving high mass loadings for micron‐scale silicon‐based materials (MSBMs) presents a significant challenge due to their relatively low tap density. In particular, during high‐rate charging, LIBs experience accelerated capacity degradation and increased safety risks. These issues can be attributed to various polarizations—ohmic, concentration, and electrochemical—that arise from kinetic mismatches within the full cell.^[^ ^33^, ^34^, ^35^, ^36^ ^]^

To address the aforementioned challenges, it is crucial to modify the silicon‐based anode both at the material level and the entire battery system, including the electrolytes, binders, and conductive agents. Several comprehensive strategies have been developed for the preparation of MSBMs, employing three main approaches: i) modification of nano‐silicon composites within a carbon matrix, ii) modification of nano‐silicon composites coated micron matrix, and iii) modification of microscale silicon composites (Figure 1b).^[^ ^30^, ^37^, ^38^, ^39^, ^40^ ^]^

Nanoscale SBMs in a micro‐matrix are fabricated by dispersing the SBMs in a well‐established conductive matrix. This multiscale structure not only provides sufficient void space to accommodate large volume changes but also enhances both electronic conductivity and ion diffusion, allowing more active materials to participate in electrochemical reactions. Moreover, multiscale particle technologies can increase the tap density of the electrode and minimize side reactions while preserving the advantages of silicon nanoparticle Si NPs. Additionally, MSBMs exhibit improved transport kinetics as the particle size approaches the nanometer scale, owing to their alloying reaction mechanism.^[^ ^41^, ^42^, ^43^, ^44^ ^]^ Therefore, nanoscale SBMs in a micro‐matrix exhibit improved cycling performance and enhanced energy/power density. However, reducing the preparation cost and production complexity of nanoscale SBMs is crucial for their industrial application. To address these challenges, researchers have explored directly depositing the nanoscale SBM layer onto a micro‐matrix (such as low‐cost commercial graphite) using techniques like chemical vapor deposition (CVD) or other methods.^[^ ^31^, ^45^ ^]^ Although this design strategy effectively reduces costs, the low specific capacity of the composite material limits the overall energy density of the device. Alternatively, another approach involves directly modifying readily available MSBMs to achieve higher energy density in LIBs.^[^ ^46^, ^47^ ^]^

Nevertheless, current strategies primarily establish a conductive network, which struggles to provide sufficient mechanical strength to prevent particle breakage and electrode structural damage during repeated volume expansions. While many previous modification strategies have focused on half‐cell electrode materials, improvements in other battery system components—such as the liquid electrolyte, solid‐state electrolyte, and binder—can also significantly contribute to overall performance enhancements (Figure 1).

In this comprehensive review, we systematically examine recent advancements in silicon‐based anodes, highlighting key breakthroughs. We begin by focusing on the structural design of silicon‐based anodes, discussing performance optimization and the lithium storage mechanism from a materials perspective. In addition, we explore the structural design and optimization of silicon‐based anodes within the context of the entire battery system, including considerations of promising liquid electrolytes, solid‐state electrolytes (SSEs), binders, and more. Furthermore, technological innovations, such as pre‐lithiation techniques to compensate for irreversible Li⁺ consumption, are emphasized for their role in enhancing the energy density and lifespan of silicon‐based full cells. Finally, a comprehensive summary of the underlying mechanisms is provided to aid in the development of high‐energy‐density devices and offer strategies for overcoming challenges in large‐scale applications.

## 2 Silicon Material Optimization

### 2.1 Modification of Nano‐Silicon Composites within a Carbon Matrix

To address the volumetric behavior and Li^+^ transport kinetics of silicon‐based anodes, nanotechnology offers a straightforward and effective strategy for optimizing alloying chemistries.^[^ ^48^ ^]^ Meanwhile, dispersing and embedding nano‐silicon materials within micron‐sized matrices, such as conductive and graphitic carbon, has proven to be an effective strategy. This approach provides a reaction substrate, enhances ion‐electron conductivity, and contributes to improved electrode stability.^[^ ^20^, ^39^ ^]^ In this section, composite electrodes consisting of Si NPs and a micro‐matrix (amorphous/graphitic carbon matrix) are systematically reviewed based on their electrochemical performance.

#### 2.1.1 Modification of Nano‐Silicon Composites in Amorphous Carbon Matrix

Amorphous carbon has been extensively studied as a 3D matrix due to its ability to enhance conductivity, provide rapid transport channels for Li^+^, buffer volume changes, and suppress the growth of SEIs on Si NPs. Typically, the 3D carbon framework is pyrolyzed from various polymers (e.g., phenolic resin, citric acid, dopamine, pyridine) to create a multilevel structure for silicon‐based composites. The following sections provide a detailed discussion of processing methods for different structural types of silicon‐based composites, including yolk–shell, core–shell, sandwich, and integrated structures.

**Figure 2:**
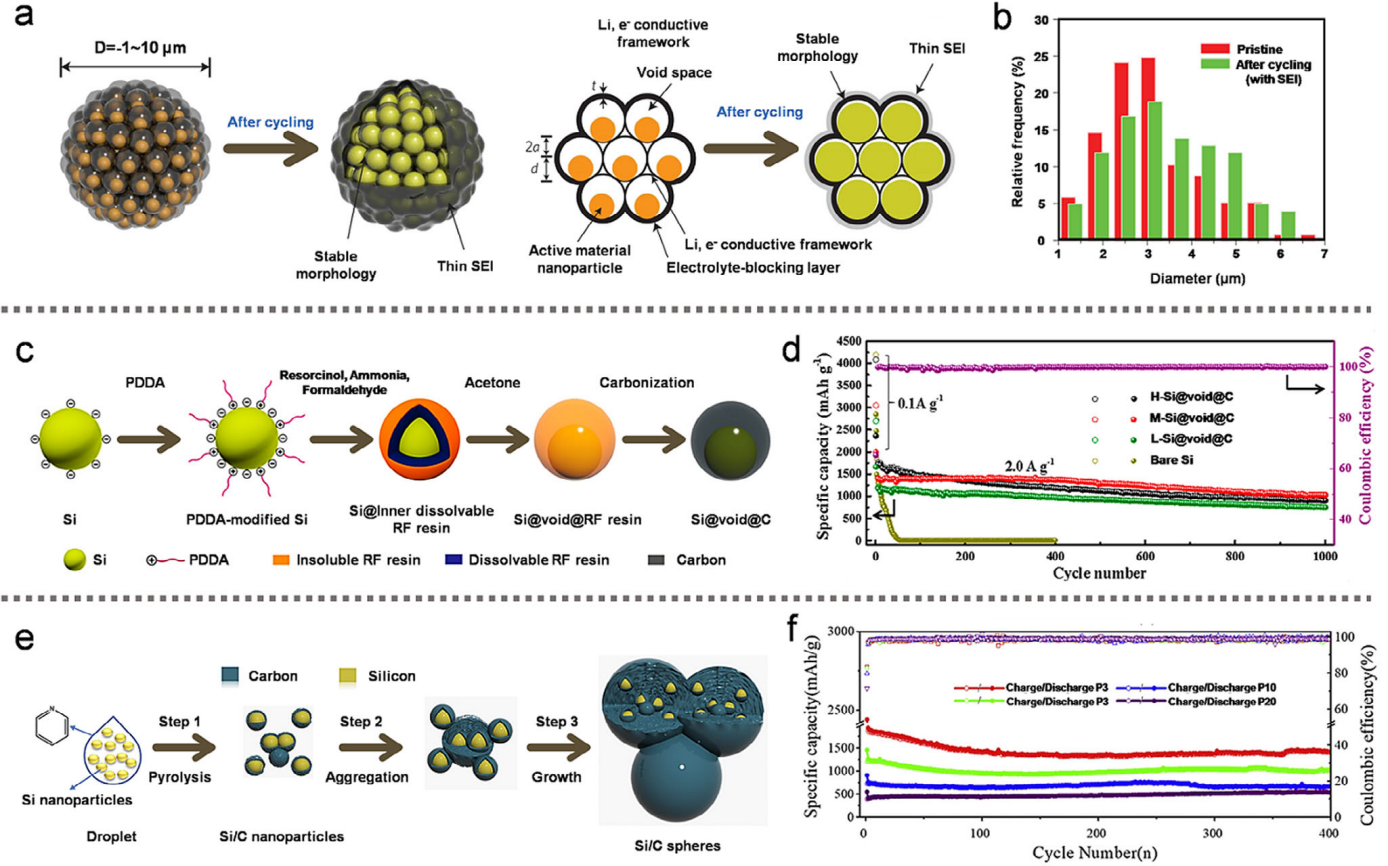
a) Evolution mechanism diagram of the Si pomegranate electrode structure during cycling. b) Statistical analysis of the Si pomegranate diameter before and after 100 cycles. (a,b) Reproduced with permission.^[^ ^50^ ^]^ Copyright 2014, Nature Publishing Group. c) Schematic illustration of the preparation process for Si@void@C anode materials using the presented templateless method. d) Cycling performance of different Si@void@C anodes at 2.0 A g^−1^. (c,d) Reproduced with permission.^[^ ^53^ ^]^ Copyright 2019, American Chemical Society. e) Schematic illustration of the formation of onion‐like Si/C spheres and f) their corresponding cycling performances. (e,f) Reproduced with permission.^[^ ^54^ ^]^ Copyright 2020, Elsevier Ltd.

To achieve longer battery life, Jung et al.^[^ ^49^ ^]^ employed an industrially scalable spray drying process to synthesize Si NP‐encapsulated porous carbon spheres. The resulting composite electrodes exhibited exceptional electrochemical performance. The spray drying process, which only required 2 seconds for the formation of each composite sphere, demonstrated the potential for high‐speed, large‐scale production of active materials. Drawing inspiration from the pomegranate structure, Liu et al.^[^ ^50^ ^]^ fabricated a hierarchical Si anode through micro‐emulsion and evaporation‐driven self‐assembly. After washing with diluted hydrofluoric acid (HF) to remove the SiO_2_ coating, the synthesized Si pomegranate composite exhibited micrometer‐sized clusters with a high tap density. The study also explored the relationship between the specific SEI film area and the number of primary Si NPs, providing insight into the determination of the optimal pomegranate particle diameter. As shown in **Figure** **2**a, the microscale pomegranate composite successfully retained the structural integrity of secondary particles and stabilized the SEI, thereby enhancing battery performance. Statistical analysis of SEI film thickness (Figure 2b) further supported the correlation between the number of pomegranate composite particles and the overall electrochemical stability. Building on this approach, Zhang et al.^[^ ^51^ ^]^ also utilized SiO_2_ as a sacrificial template to fabricate a granadilla‐like, porous Si/C microsphere structure via spray drying followed by high‐temperature carbonization.

In contrast to the previously mentioned approaches, a green and simple method was developed by removing the sacrificial template (calcium carbonate) using diluted hydrochloric acid (HCl), rather than HF.^[^ ^52^ ^]^ The resulting double‐carbon‐shell‐structured Si/C microspheres featured an interconnected, granadilla‐like carbon framework with internal void spaces, effectively enhancing electrode stability and providing robust protection. Wang et al.^[^ ^53^ ^]^ also proposed a facile, template‐free approach for the preparation of Si@void@C materials by controlling the growth kinetics of resorcinol‐formaldehyde (RF) resin (Figure 2c). This method is environmentally friendly, scalable, and offers flexible control over key parameters in the production of Si@void@C materials. By adjusting the RF resin growth kinetics on the surface of Si NPs, a higher polymerization degree was achieved in the outer layer, while the inner layer exhibited a lower degree of polymerization. The RF resin served both as the carbon source and the sacrificial layer. The Si@void@C anode demonstrated excellent cycling stability and superior rate performance (Figure 2d). In Figure 2e, a simple one‐step injection pyrolysis method was developed to prepare onion‐like Si/C anodes.^[^ ^54^ ^]^ In this approach, Si NPs were encapsulated with nitrogen‐doped carbon shells using pyridine as the carbon and nitrogen source. The onion‐like carbon layer, ≈10 nm thick, improved conductivity and alleviated stress in all directions, effectively preserving the structural integrity of the electrode over extended cycling. As a result, the anode material exhibited outstanding lithium‐storage performance, delivering a capacity of 1391 1391 mA h g^−1^ after 400 cycles at 0.2 A g^−1^ in Figure 2f.

**Figure 3:**
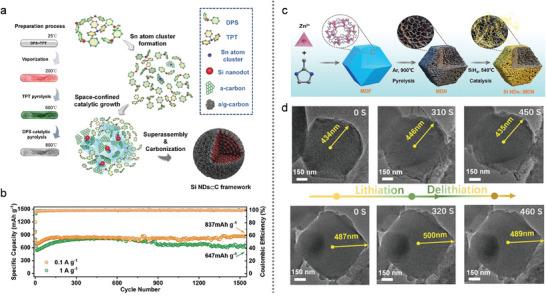
The schematic illustrations of a) the synthetic procedure for Si NDs@C and b) their cycling performance at 0.1 and 1 Ag^−1^. (a,b)Reproduced with permission.^[^ ^56^ ^]^ Copyright 2020, Wiley‐VCH. c) The formation process of the Si NDs@MDN composite, and d) in situ TEM images of individual Si NDs⊂MDN samples during a lithiation–delithiation cycle. (c,d) Reproduced with permission.^[^ ^57^ ^]^ Copyright 2022, Wiley‐VCH.

As the size of Si particles decreases from the nanoscale to the sub‐nanoscale (≈1 nm), subnano‐sized Si anodes experience significantly reduced strain, enabling them to tolerate large dimensional changes without fragmentation. Additionally, the smaller particle size improves the lithium‐ion transport kinetics. However, subnano‐sized Si is prone to air instability due to excessive oxidation, as Si readily forms a native oxide layer (SiO_2_) when exposed to air.^[^ ^55^ ^]^ To address this issue, incorporating subnano‐sized Si with other elements has emerged as a rational strategy. In **Figure** **3**a, Chen et al.^[^ ^56^ ^]^ introduced a novel super‐assembly approach for fabricating Si nanodots embedded in a carbon (Si NDs@C) framework via a co‐pyrolysis method. To prevent the formation of SiC and facilitate the formation of Si NDs, Sn atomic clusters derived from triphenyltin hydride pyrolysis were used as catalysts to alter the reaction pathway. The Sn clusters broke the Si‐C bonds of diphenylsilane (DPS) and promoted the graphitization of the carbon framework at lower temperatures, resulting in Si NDs. In Figure 3b, the Si NDs@C framework electrode exhibited a high reversible capacity of 837 mA h g^−1^ at 0.1 A g^−1^ for 1500 cycles over 1500 cycles. The catalytic mechanism of DPS pyrolysis by Sn atomic clusters was also simulated to validate the catalytic process. These results demonstrated that Sn atomic clusters significantly lowered the energy barrier for Si formation at 800 °C, whereas SiC formation occurred without the Sn catalyst. In Figure 3c, Yang et al.^[^ ^57^ ^]^ prepared a Si/C composite (denoted Si NDs@MDN) by embedding Si NDs in MOF‐derived nanoreactors (MDNs) within a porous carbon matrix. They confirmed that the Si NDs⊂MDN electrode exhibited a zero‐strain property and high structural stability via an in situ TEM characterization. As shown in Figure 3d, the real‐time volume change of Si NDs⊂MDN was only ≈2.76% during the lithiation–delithiation cycle. The Si NDs@MDN anode, with a high Si loading (30.60 wt.%), demonstrated a high ICE of 87.94% and excellent capacity retention of 90.1% after 1000 cycles at 1A g^−1^.

Oxidation of Si is an effective strategy for enhancing the cycling performance of crystalline Si anodes. By adjusting the oxygen content in SiO_x_, the cycle stability of the electrode can be improved. Generally, as the oxygen content increases, irreversible products such as lithium silicates and Li₂O can help buffer volume expansion and reduce crack formation, thus improving the cycle stability of SiO_x_ to some extent. However, these irreversible products also consume a portion of the available Li⁺ in the full cell, which leads to a decrease in ICE and a reduction in the actual energy density.^[^ ^58^, ^59^ ^]^ Furthermore, the formation of chemically stable Li₄SiO₄ hinders the release of Li⁺, leading to limited electrode kinetics, increased polarization, and degraded rate performance.

Currently, the crystal structure of SiO_x_ remains incompletely understood due to its complex composition and structure. It is generally accepted that SiO_x_ is a microstructural mixture of Si and amorphous SiO₂, with numerous internal connections.^[^ ^60^, ^61^ ^]^ Commercial SiO_x_ is typically produced by evaporating and depositing SiO₂ and Si. However, at high temperatures, SiO_x_ undergoes disproportionation, decomposing into Si and SiO₂ (2SiO_x_ → Si + SiO₂_x_). After disproportionation, the growth of Si within SiO₂ leads to a two‐phase reaction, which increases the risk of SiO_x_ particle fracture. Therefore, overcoming these challenges in SiO_x_ particles is essential for advancing their commercialization and industrial applications.

In 2001, Gao et al.^[^ ^62^ ^]^ demonstrated the electrochemical activity of commercially available SiO₂ NPs with an approximate size of 7 nm toward lithium. Although a specific capacity of around 400 mAh g⁻¹ was achieved, nanostructured SiO₂ still falls significantly short of the theoretical capacity of 1965 mAh g⁻¹. Notably, SiO_x_ is composed of nano‐sized Si domains embedded within a SiO₂ matrix. Building on the concept of Si modification, smaller SiO_x_ particles were uniformly dispersed in a carbon matrix to enhance their electrochemical performance.^[^ ^63^, ^64^ ^]^ This approach seeks to boost the electrochemical activity of SiO_x_ while mitigating the stress caused by the volume expansion of the active material. Mai et al.^[^ ^65^ ^]^ engineered monodisperse SiO_x_/C microspheres through the simultaneous condensation of organosilica and resorcinol‐formaldehyde polymer. In this structure, SiO_x_ ultrafine nanodomains (<2 nm) were uniformly dispersed within the carbon matrix. By leveraging these unique structural features, the SiO_x_/C composite exhibited a reversible capacity of 689 mA h g^−1^ with a capacity retention of 91.0% after 400 cycles. Additionally, they introduced a tri‐component co‐assembly approach to fabricate ultrafine SiO₂/resin nanospheres.^[^ ^66^ ^]^ After spray drying and carbonization, the resulting pomegranate‐like SiO_x_/C microspheres demonstrated a discharge capacity of 1024 mA h g^−1^ after 200 cycles. Another synthesis method involved preparing SiO_x_/C microspheres in sealed vessels under high temperature and pressure by heating a liquid mixture of polydimethylsiloxane and hexane.^[^ ^67^ ^]^ The homogeneous dispersion of SiO_x_ and free carbon effectively mitigated volume expansion and improved electrical conductivity. Consequently, the SiO_x_/C microspheres exhibited a notable areal capacity of 2.6 mAh cm^−2^ over 400 cycles, even at a high mass loading of 3.0 mg cm^−2^.

**Figure 4:**
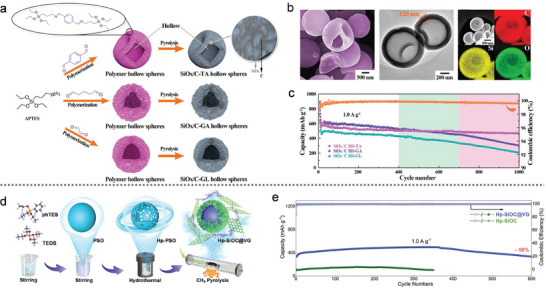
a) Schematic illustration of the synthesis procedure for SiO_x_ nanoclusters encapsulated in a hollow carbon shell. b) SEM and TEM images of SiO_x_/C HS‐TA, along with HAADF‐STEM imaging and the elemental distribution maps for carbon (C), oxygen (O), and silicon (Si). c) Long‐term cycling performance of SiO_x_ nanoclusters encapsulated in a hollow carbon shell at 1.0 A g^−1^. (a–c) Reproduced with permission.^[^ ^68^ ^]^ Copyright 2021, Wiley‐VCH. d,e) Fabrication process for rambutan‐like Hp‐SiOC@VG and their corresponding long‐term cycling performance at 1.0 A g^−1^. (d,e) Reproduced with permission.^[^ ^69^ ^]^ Copyright 2023, Wiley‐VCH.

In **Figure** **4**a, Du et al.^[^ ^68^ ^]^ synthesized SiO_x_/C hollow spheres using a method that combined molecular polymerization with pyrolysis. This process involved the formation of polymer hollow spheres (PHSs) through the rapid condensation of amine‐terminated organoalkoxysilane (3‐aminopropyltriethoxysilane) and dialdehyde molecule droplets. Various dialdehyde molecules, such as terephthalaldehyde (TA), glutaraldehyde (GA), and glyoxal (GL), were used as crosslinkers, allowing precise control over the morphology and carbon content of the PHSs. Figure 4b illustrates the uniform integration of SiO_x_ nanoclusters within a hollow carbon shell. As a result, the SiO_x_/C HS‐TA anodes exhibited exceptional cycling stability, with only 8.3 mAh g⁻¹ of capacity fading per 100 cycles at 1.0 A g⁻¹ (Figure 4c). Furthermore, in Figure 4d, rambutan‐like, vertical graphene‐coated hollow porous Si oxycarbide (Hp‐SiOC@VG) spherical particles were fabricated using hydrothermal treatment combined with CH_4_ pyrolysis.^[^ ^69^ ^]^ This method increased the reversible SiO_3_C and SiO_2_C_2_ structural units, enhancing Li^+^ adsorption capacity compared to the SiOC_3_ counterpart. Consequently, the designed Hp‐SiOC@VG demonstrated remarkable cycling stability, with a capacity retention of 98% after 600 cycles at 1.0 A g⁻¹ (Figure 4e).

#### 2.1.2 Modification of Nano‐Silicon Composites in Graphitic Carbon Matrix

Compared to amorphous carbon, graphitic carbon offers superior electrical conductivity and mechanical elasticity, effectively mitigating volume stress during cycling.^[^ ^70^, ^71^, ^72^, ^73^ ^]^ Moreover, the high raw material costs, low yield, and side reactions associated with organic‐derived amorphous carbon present significant barriers to its industrial application. By replacing amorphous carbon with graphitic carbon, both the cycling stability and transport kinetics of the electrode can be enhanced. Currently, various materials—such as carbon NPs,^[^ ^74^ ^]^ graphene,^[^ ^75^ ^]^ carbon nanotubes (CNTs),^[^ ^76^, ^77^ ^]^ and graphite flake^[^ ^78^ ^]^—are being utilized to address these challenges, thanks to their exceptional mechanical flexibility, chemical stability, and electrical conductivity. This strategic shift holds considerable potential for advancing the practical application of carbon‐based materials in energy storage systems.

Achieving effective interface contact by directly mixing these carbon materials with Si remains challenging. Organic carbon sources are required to mediate and suppress the repeated volume expansion during cycling. Pitch‐pyrolytic carbon (pitch‐C), a blend of amorphous and graphitic carbon, plays a critical role in establishing strong interfacial adhesion and ensuring conductivity between the silicon‐based material and the graphitic carbon matrix.^[^ ^79^ ^]^ In this context, SiO_x_ particles were dispersed and anchored within multicomponent carbon materials to form SiO_x_/GO/flake graphite (SiO_x_/GO/FG) composites.^[^ ^80^ ^]^ The shrinkage of asphalt adsorbed in SiO_x_/C at high temperatures facilitated the restoration of the original structure of artificial graphite, leading to a compact structure with a high tap density. This optimized design promoted the formation of a stable SEI layer, enhanced electrical conductivity, and preserved the structural integrity of the SiO_x_ electrode. As a result, the SiOx/GO/FG anodes exhibited a high ICE of 89.5% and demonstrated excellent cycling stability, with ≈90% capacity retention after 500 cycles. Additionally, the core–shell plasma nano‐silicon@carbon (PNSi@C) composite was integrated onto thin milled flake graphite (MFG) sheets, each with a thickness of 150 nm, using a simple spray‐drying method.^[^ ^81^ ^]^

Subsequently, Guo et al.^[^ ^82^ ^]^ successfully developed Si/C microspheres with a compact nano/microstructure under high‐temperature and high‐pressure conditions. In this novel architecture, Si NPs anchored to flake graphite were uniformly dispersed and self‐assembled into microspheres with the assistance of soft, adhesive pitch. The encapsulation of Si within these dense carbon microspheres enhanced their compressive properties under high rolling pressure while maintaining a low specific surface area. This combination facilitated the formation of a stable SEI film, thereby minimizing side reactions. Furthermore, the incorporation of flake graphite and pitch‐derived carbon formed a robust 3D carbon framework, which ensured the electrochemical activity of the Si NPs. As a result, the Si/C microspheres exhibited a high tap density of 1.0 g cm^−3^ and demonstrated impressive cycling stability, retaining an areal capacity of 4 mA h cm^−2^.

**Figure 5:**
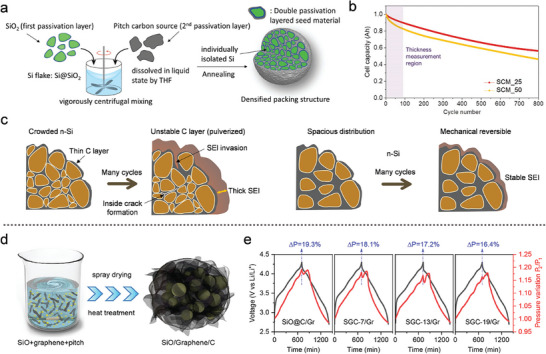
a) Schematic illustration of the synthetic procedure for Si@SiO_2_@C micro‐particles. b) 1 Ah full‐cell test of the SCM electrode at 25 and 50  C. c) Mechanical degradation and irreversible reaction effects in SCM and u‐SCM during cycling. (a–c) Reproduced with permission.^[^ ^83^ ^]^ Copyright 2020, Wiley‐VCH. d) Schematic illustration of the fabrication process for SGC composites. e) Voltage–time and pressure–time plots for different SGC composites during the charging‐discharging cycles. (d,e) Reproduced with permission.^[^ ^75^ ^]^ Copyright 2021, Elsevier Ltd.

To address significant volume fluctuations and achieve high electrode density, Si‐based anodes without voids were designed using FEM calculations. The micron‐sized double passivation Si/C composite (µ‐SCM) featured a highly densified structure and a restrictive lithiation state, as shown in **Figure** **5**a.^[^ ^83^ ^]^ As a result, a 1 Ah pouch‐type full cell, evaluated under industrial conditions (≈3.75 mA h cm^−2^ and ≈1.65 g cm^−3^), demonstrated superior cycling stability, surpassing 800 cycles (Figure 5b). Compared to SCM, µ‐SCM effectively mitigated volume changes due to its enhanced mechanical properties and increased bulk density during cycling, as depicted in Figure 5c. In Figure 5d, a mechanically durable carbon layer was applied to encapsulate SiO, forming SiO/Graphene/C (SGC) composites.^[^ ^75^ ^]^ An in situ electrochemical‐pressure test was conducted, with the corresponding voltage‐time and pressure‐time plots shown in Figure 5e. The relative pressure variation (Δ*P*) was calculated using the formula Δ*P* = (*P*_2_ − *P*_1_)/*P*_1_×100%, where *P*_1_ and *P*_2_ represent the pressure at the start and end of the charging process, respectively. These results demonstrated that the graphene‐coupled pitch‐pyrolytic carbon outer layer provided high electrical conductivity and compressive density while establishing a mechanical‐inhibition interaction to restrict the volume expansion of alloy anodes. As a result, the SGC anode, containing 19 wt.% graphene, exhibited enhanced cycling stability with a capacity retention of 86.2% after 500 cycles.

Furthermore, heteroatom‐doped carbon is used to enhance the bonding strength between the active components of the battery and improve overall electrode conductivity. Inspired by the structure of a watermelon fruit, hierarchical buffer‐structured Si/C microspheres were designed by dispersing carbon‐coated nano‐Si in a flake graphite matrix, followed by the deposition of a complete carbon shell.^[^ ^78^ ^]^ The hierarchical buffer structure and optimized size distribution were engineered to accommodate significant volume fluctuations and stabilize the SEI layer in densely packed electrode materials. This optimized design enabled the watermelon‐inspired Si/C microspheres to achieve superior capacity retention of 75.2% after 500 cycles at 0.5 C.

In another design, Si NPs were embedded in a buffer carbon matrix to create a mixed 3D conducting network through a spray‐drying and pyrolysis process.^[^ ^84^ ^]^ The resulting Si/C granules, with a high mass loading of 8.5 mg cm^−2^, exhibited excellent electrochemical performance. The incorporation of graphitic carbon further enhanced the electrical conductivity and mechanical flexibility of the mixed carbon skeleton. Additionally, a novel “Janus shell” structure was developed by encapsulating Si, designed to form a stable SEI layer. In this structure, the nanopores in the loosely stacked graphene shell were mostly sealed by the conformal carbon outer shell.^[^ ^85^ ^]^ Benefiting from this “Janus shell” design, the synthesized anodes demonstrated superior capacity retention of 88.5% after 400 cycles at 2 A g^−1^.

**Figure 6:**
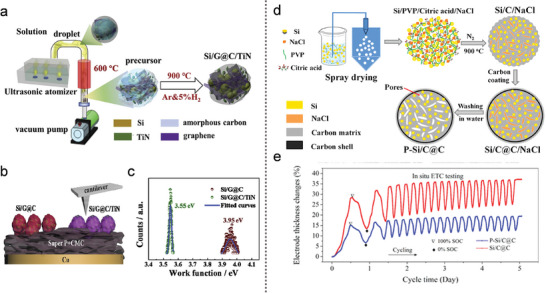
a) Schematic illustration of Si/G@C/TiN composites. b) Schematic of the electrode used for work function measurements, and c) Calculated work functions of Si/G@C and Si/G@C/TiN composites after 50 cycles. (a–c) Reproduced with permission.^[^ ^86^ ^]^ Copyright 2020, Elsevier Ltd. d) Schematic illustration of the synthesis process for P‐Si/C@C composites and e) the in situ electrode thickness change measurements of P‐Si/C@C and Si/C@C. (d,e) Reproduced with permission.^[^ ^87^ ^]^ Copyright 2022, American Chemical Society.

As illustrated in **Figure** **6**a, Li et al.^[^ ^86^ ^]^ developed Si/graphene@carbon composites embedded with TiN (Si/G@C/TiN), incorporating polyvinyl pyrrolidone as a carbon‐coating layer to effectively accommodate the significant volume expansion of Si particles during cycling. The TiN network, embedded within the porous carbon framework, not only facilitated enhanced electron transfer but also promoted uniform heat distribution within the electrode. To systematically investigate the physical and chemical properties of the SEI film on the anode materials, surface potential maps of Si/G@C and Si/G@C/TiN electrodes were obtained using Kelvin probe atomic force microscopy, as shown in Figure 6b. Through calculation and fitting analysis, it was observed that the lower work function of Si/G@C/TiN facilitated improved electron transfer at the composite surface, thereby inhibiting excessive SEI film growth (Figure 6c). The Si/G@C/TiN electrodes demonstrated significantly enhanced cycling stability, maintaining a capacity of 660 mAh g⁻¹ at 10 A g⁻¹ and 776.5 mAh g⁻¹ at 5 A g⁻¹ after 400 cycles.

Interestingly, micro/nanostructured, pore‐rich Si/C microspheres with an outer robust carbon shell (denoted P‐Si/C@C) were synthesized using inorganic salts as pore‐forming agents (Figure 6d).^[^ ^87^ ^]^ The enhanced structural stability of P‐Si/C@C can be attributed to the combined effect of the inner voids and the durable outer carbon shell, which together reinforce the integrity of the entire structure. In Figure 6e, the changes in electrode thickness for Si/C@C and P‐Si/C@C anodes were monitored over the initial 20 cycles, spanning ≈5 days. During the first cycle, P‐Si/C@C exhibited a lower electrode swelling of 15.2%, compared to 28.4% for Si/C@C. This trend continued through the 20th cycle, underscoring the superior cycling stability and safety of Si‐based LIBs. As a result, the P‐Si/C@C composite demonstrated an ICE of 89.8% and excellent capacity retention of 87.1% after 820 cycles at 1 A g^−1^. Notably, an 18650‐type P‐Si/C@C//NCA cylindrical cell exhibited remarkable cycling stability, retaining 81.4% of its capacity after 1200 cycles at 3.2 A g^−1^.

Moreover, the direct deposition of a graphitic carbon layer onto Si‐C composites has proven to be an effective approach, though the associated costs remain a consideration. A unique nano/microstructured Si‐C composite material was synthesized using a scalable microemulsion method, where low‐cost corn starch served as a biomass precursor, which was then carbonized under a C_3_H_6_ atmosphere.^[^ ^88^ ^]^ The resulting Si NPs were embedded in micron‐sized amorphous carbon spheres and encapsulated by a thin graphitic carbon layer. This Si‐C hybrid composite, featuring a double carbon matrix, provided high electronic conductivity, effectively ensuring strain relaxation and maintaining structural integrity during multiple lithiation–delithiation cycles. The Si‐C hybrid anode exhibited excellent cycling stability, with 80% capacity retention over 500 cycles, and demonstrated fast charge‐discharge capability, completing a cycle in just 12 minutes.

**Figure 7:**
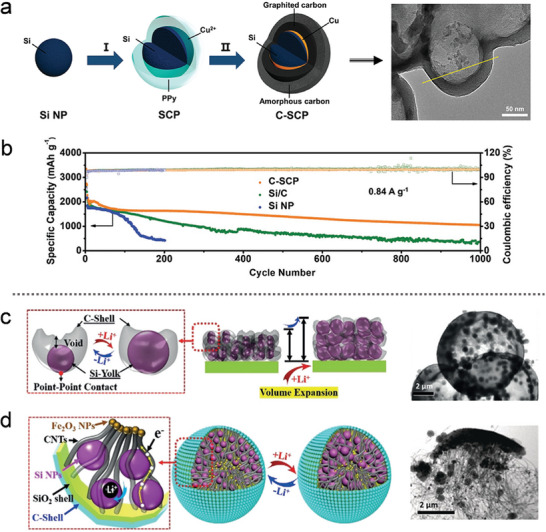
a) Schematic illustration of the synthetic procedures for C‐SCP products, and b) long‐term cycling performance of Si NP, Si/C, and C‐SCP at 0.84 A g^−1^. (a,b) Reproduced with permission.^[^ ^89^ ^]^ Copyright 2020, Wiley‐VCH. Schematic illustration and TEM images of c) the existing yolk–shell Si/C structure and d) the proposed new yolk–shell Si/C structure. (c,d) Reproduced with permission.^[^ ^76^ ^]^ Copyright 2019, Wiley‐VCH.

The graphitization degree and morphology of organically derived carbon can be significantly influenced by the introduction of metal elements. To address the high interfacial energy between Si and C resulting from lattice mismatch, a metal layer was applied to the Si surface to enhance interfacial kinetics. Copper (Cu), known for its excellent conductivity and ductility, was chosen as the exclusive metallic current collector for the anode. Cu facilitates lithium‐ion transfer without forming alloys, enabling the development of a scalable conductive Cu interface (CCI) through a self‐assembly carbothermic process.^[^ ^89^ ^]^ In the Si/Cu polypyrrole (SCP) composite (**Figure** **7**a), a significant amount of Cu was incorporated into the polypyrrole (PPy) layer, due to strong coordination interactions between the delocalized electrons in PPy and Cu^2^⁺. The C‐SCP product was then derived from the SCP composite. During this process, metallic Cu tended to diffuse toward the Si/C interfaces under the high temperatures of carbothermic treatment. Thanks to its multi‐layered structure, the Si with CCI achieved a high areal capacity of 4.78 mAh cm^−2^ after 280 cycles. As shown in Figure 7b, the C‐SCP electrode exhibited an exceptionally low fading rate of 0.068% per cycle, maintaining a discharge capacity of 1050 mAh g^−1^ at the 1000th cycle.

Research suggests that the existing yolk–shell Si/C structures, as shown in Figure 7c, provide sufficient void space to accommodate the volume expansion of Si NPs. However, the limited contact area between the Si yolk and the C‐shell diminishes the overall electrical conductivity of the electrode. To address this issue, Zhao et al.^[^ ^76^ ^]^ reported a novel yolk–shell structured Si/C composite (YS‐Si/C) with a high tap density, achieved by bridging a conductive carbon nanotube (CNT) “highway” between the inner Si yolk and the outer shell, as illustrated in Figure 7d. The CNTs were synthesized using iron oxide (Fe₂O₃) as a catalyst. In the YS‐Si/C composite, the carbon‐coated, rigid SiO_2_ outer shell and the CNT network effectively constrained the volume changes of the Si yolks and enhanced the overall conductivity of the electrode. As a result, the YS‐Si/C anode demonstrated impressive cycling stability, achieving a reversible capacity retention of 95% after 450 cycles, with an ICE of 86.9%.

Si NPs were dispersed in an amorphous carbon matrix to form multi‐layer hierarchical structures that provide a conductive network and buffer space, enhancing both electron transport and accommodating volume expansion during cycling. However, the relationship between achieving high tap density and maintaining a hierarchical porous structure remains complex. While high tap density can improve the overall energy density, it also increases the risk of structural degradation, particularly with significant volume changes in Si during cycling. The challenge lies in balancing these factors to maximize both energy density and long‐term stability. Additionally, accurately controlling the thickness, porosity, and uniformity of the carbon layer on the Si NPs is crucial to prevent issues such as poor cycling stability and capacity fading. The carbon coating should be sufficiently thick to serve as a protective layer that buffers volume changes, but it should not be so uneven or thick that it restricts the lithiation–delithiation kinetics or increases internal resistance. Moreover, the agglomeration of graphitic carbon materials is a critical issue that can negatively affect the electrochemical performance and stability of Si‐based anodes. Advanced dispersion techniques, surface modifications, hybrid structures, and optimized binder systems can mitigate agglomeration, improve material integration, and enhance long‐term performance. Furthermore, the excessive formation of silicon‐carbon (Si‐C) bonds at high temperatures can compromise cycling performance due to its limited ionic conductivity and the mechanical stress it generates during cycling. For commercial viability, optimized anodes must demonstrate long‐term cycling stability under real‐world conditions, particularly at high loadings and commercial scales.

### 2.2 Modification of Nano‐Silicon Composites Coated Micron Matrix

Si and graphite composite electrodes (Si/G) have attracted significant attention as promising candidates for high‐performance LIBs in industrial applications.^[^ ^90^, ^91^, ^92^, ^93^, ^94^ ^]^ However, challenges such as high production costs, substantial volume expansion, and poor electronic conductivity have hindered the widespread adoption of Si‐based anodes as replacements for commercial graphite. To address these challenges, Si/G composite electrodes with optimized ratios have been designed as effective countermeasures. Obrovac et al.^[^ ^95^ ^]^ found that blending Si particles with smaller‐sized graphite could enhance cycling performance. However, the large volume expansion of Si particles often leads to degradation of the electrode microstructure and the electrical network, resulting in rapid performance decline.^[^ ^96^ ^]^ To mitigate the volume change of Si particles and preserve a stable conductive network, graphene, as an ideal 2D support material, has been extensively utilized.^[^ ^94^ ^]^ To reduce rapid capacity fading, Si/graphite composites were homogeneously encapsulated within a graphene scaffold, achieving a high capacity retention of 90% after 100 cycles at 372 mA g^−1^.^[^ ^97^ ^]^

Maintaining the structural integrity of Si and graphite during cycling remains a significant challenge. In contrast, commercially available organic materials such as sucrose, wheat flour, pitch, polydopamine, citric acid, and styrene‐acrylonitrile copolymer (SAN), among others, have been utilized as carbon sources. These materials facilitate the incorporation of Si NPs with nanographene, forming an interconnected structure that enhances cycling stability.^[^ ^98^, ^99^, ^100^, ^101^ ^]^ Building upon previous work, flake‐shaped Si plates within the SAN‐derived carbon matrix were further encapsulated by pyrolyzed carbon and natural graphite.^[^ ^102^ ^]^ However, the self‐assembled Si/C materials, with a particle size range of 15–50 µm, exhibited a relatively low capacity retention of only 428.1 mA h g^−1^ after 100 cycles at 0.1 mA g^−1^.

To replace commercial graphite, a graphite‐alloy composite electrode must meet several key industrial criteria: high areal capacity loading (≥3.5 mAh cm^−2^)), high capacity retention (>80% over ≈500 cycles), high tap density (≥1.6 g cm⁻^3^), and limited binder content (≤3 wt.%). Achieving a low electrode swelling ratio during cycling is also crucial to meet high‐energy demands. Uniform and thin Si coating layers can be effectively produced using CVD, with silane serving as the precursor gas. For example, Su et al.^[^ ^93^ ^]^ employed the CVD method to grow Si/C microrods on the surface of natural graphite (NG), achieving a specific capacity of 562.0 mAh g^−1^ at 50 mA g^−1^. In another approach, a novel Si‐on‐graphite (Si@G) composite was synthesized using a double coating strategy—pitch‐derived carbon and a polymer coating—to achieve a uniform distribution of Si NPs on graphite MPs^[^ ^103^ ^]^ The double coatings provided sufficient space to accommodate volumetric expansion of the entire electrode. The polymer coating may serve as an artificial SEI layer to mitigate the formation of a natural SEI of higher charge‐transfer resistance, which can improve cycle stability and rate performance.

**Figure 8:**
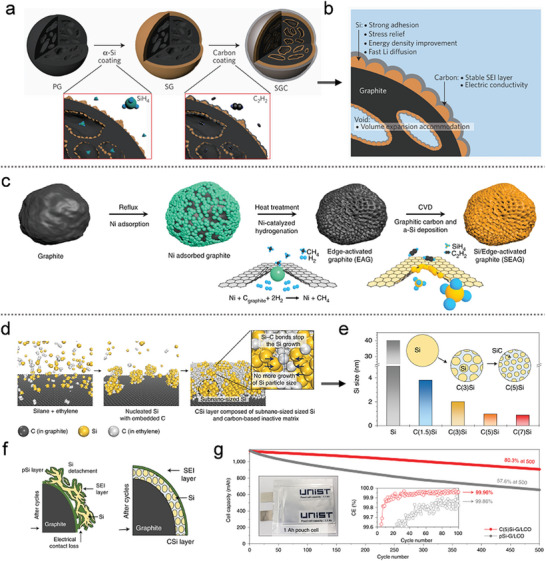
a) Schematic illustration of the fabrication process of SGC hybrid particles. b) Corresponding cross–sectional schematic view of the SGC composite. a,b) Reproduced with permission.^[^ ^104^ ^]^ Copyright 2016, Nature Publishing Group. c) Schematic depiction of the fabrication procedures for SEAG composites. (c) Reproduced with permission.^[^ ^32^ ^]^ Copyright 2017, Nature Publishing Group. d) Schematic diagram illustrating the fabrication process of subnano‐sized Si/graphite composites. e) Calculated crystallite size of Si in various subnano‐sized Si/graphite composites. f) Schematic representation of lithium‐ion behavior in pSi‐G and subnano‐sized Si/graphite (C(5)Si‐G) composites, and g) the corresponding long‐term cycling in full‐cell configurations. (d–g) Reproduced with permission.^[^ ^105^ ^]^ Copyright 2021, Nature Publishing Group.

As shown in **Figure** **8**a, Cho et al.^[^ ^104^ ^]^ developed a novel architecture for Si nanolayer‐embedded graphite/carbon (SGC) hybrids. The uniform Si nanolayers, with a thickness of <20 nm, were deposited on the graphite surface to mitigate induced stress and facilitate rapid lithium‐ion transport, leading to a significant enhancement in energy density (as depicted in Figure 8b). The subsequent carbon coating further stabilized the formation of the SEI layer while improving the electrode's electrical conductivity. This design resulted in an ICE of 92% and long cycle life under industrial electrode composition conditions (with an electrode density exceeding 1.6 g cm^−3^ and areal capacity loading exceeding 3.3 mAh cm^−3^).

Building on this approach, a novel Si‐graphite composite was proposed by incorporating a uniformly implanted amorphous Si nanolayer and edge‐site‐activated graphite (EAG), as shown in Figure 8c.^[^ ^32^ ^]^ Due to the catalytic reaction (Ni + Cgraphite + 2H_2_ → Ni + CH_4_), EAG achieved a double reactive surface area compared to pristine graphite, thereby improving the mass‐transfer kinetics of lithium ions. Hence, the amorphous‐Si nanolayer‐coated edge‐plane activated graphite (SEAG) composite in Figure 8c was prepared by depositing a protective graphitic carbon shell and a Si layer of ≈18 nm. This architecture successfully achieved higher energy density and withstood mechanical pressure due to the sturdy inner core. Consequently, the hybrid anode exhibited predominant fast‐charging behavior with an ICE of 93.8%. In addition, the SEAG//LCO full‐cell demonstrated higher volumetric energy density (1060 Wh L^−1^) with 1.5 times faster charging compared to that of a conventional graphite anode.

To further optimize the Si layer structure on the graphite surface, a growth inhibition mechanism was proposed based on theoretical simulations, aiming to prevent the formation of Si‐Si bonds by promoting the formation of multiple Si‐C bonds, as illustrated in Figure 8d.^[^ ^105^ ^]^ The subnano‐sized Si composite was deposited onto a graphite substrate through the thermal decomposition of complex gases (SiH_4_ and C_2_H_4_), where the growth of Si clusters was suppressed, resulting in subnano‐sized Si particles embedded in a stable dual matrix of Si carbide and amorphous carbon. In contrast, the pristine Si‐graphite (pSi‐G) composite was synthesized by the thermal decomposition of pure SiH₄ gas. The crystallite size of Si in each sample was calculated using the Scherrer equation and is shown in Figure 8e. The subnano‐sized Si composite exhibited a significantly smaller crystallite size (ranging from 0.87 to 3.8 nm) compared to the pSi‐G composite, which had a crystallite size of ≈40 nm. Benefiting from this structure design (Figure 8f), the subnano‐sized Si composite on the graphite substrate demonstrated improved electrode stability and the formation of a stable SEI layer, as opposed to the less stable SEI formed when Si was directly loaded onto graphite. As a result, the subnano‐sized Si/graphite composite anode achieved a high ICE of 93.1% and excellent cycling stability, retaining over 80% of its capacity after 500 cycles. Furthermore, the fabricated energy storage system (with a total capacity of 103.2 kWh) comprising 110 Ah full‐cells exhibited outstanding long‐term cycling performance, maintaining 91% of its capacity after 2875 cycles, as shown in Figure 8g.

In comparison to other carbon coating methods, pitch‐derived carbon coatings are particularly notable for their ability to withstand significant volume expansion due to their superior mechanical strength. Petroleum pitch, a cost‐effective carbon source, has been widely used for this purpose. Leveraging these advantages, compared with other carbon coating methods, pitch‐derived carbon coating stands out due to its ability to endure severe volume expansion owing to superior mechanical strength. Petroleum Pitch is well‐known as a cost‐effective carbon coating source. Considering these advantages, Cho et al.^[^ ^106^ ^]^ developed a straightforward and low‐cost method to apply pitch coatings to high‐capacity Si nanolayer‐embedded graphite (denoted SGCpitch). This coating not only enhanced the electrical conductivity of the composite but also effectively mitigated volume expansion during electrochemical lithiation. As a result, the SGCpitch anode demonstrated improved cycling stability, with a capacity retention of 81.9% after 200 cycles.

**Figure 9:**
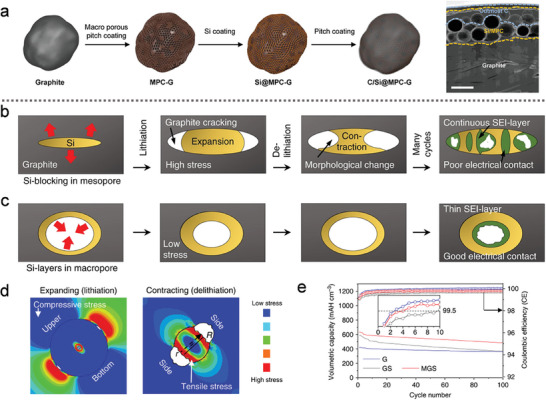
a) Schematic illustration of the fabrication procedures of C/Si@MPC‐G composites and the corresponding SEM image. (a) Reproduced with permission.^[^ ^45^ ^]^ Copyright 2020, Wiley‐VCH. b,c) Schematic illustrations of the lithiation–delithiation behavior of the mesopore Si‐blocking and the macropore Si‐blocking. d) The lithiation–delithiation process of the mesopore Si‐blocking based on finite element results and e) the cycling performance and Coulombic efficiency of G (graphite), GS (Si‐layer‐coated G), and MGS in Full‐cell. (b–e) Reproduced with permission.^[^ ^31^ ^]^ Copyright 2019, Nature Publishing Group.

However, these Si‐graphite composites were limited by the low Si content. Excessive Si deposition on graphite could lead to performance degradation due to the high strain induced during cycling. To overcome this challenge, Cho's group^[^ ^45^ ^]^ proposed a calendering‐compatible Si‐graphite anode, fabricated through mechanical agitation and CVD, as illustrated in **Figure** **9**a. This approach involved the construction of an ultrathin Si‐coated macroporous carbon‐graphite architecture (C/Si@MPC‐G) with a carbon outer layer, which maximized volumetric energy density. The non‐filled porous structure of the composite enabled it to withstand high mechanical pressure without fracturing, while also accommodating the volumetric changes of the Si nanolayer during cycling. The resulting composite exhibited an impressive areal capacity of over 3.6 mA h cm^−2^ while maintaining a nonactive material content of <5 wt.%, demonstrating both high efficiency and scalability for practical applications.

In the lithiation–delithiation process, Si‐blocking within the graphite mesopores (GS) experiences extreme stresses, leading to the formation of cracks in the graphite and the fracturing of Si particles, as illustrated in Figure 9b.^[^ ^31^ ^]^ This phenomenon can directly damage electrical contacts, resulting in the continuous formation of the SEI layer. In contrast, Si‐layers within graphite macropores (MGS), which provide more space for expansion, help to alleviate stress concentration and better accommodate volume changes. This leads to the formation of a relatively stable SEI layer and improved electrical contact, as depicted in Figure 9c. After the thermal decomposition of ethylene gas, Si‐layers with a thickness of at least 7 nm were uniformly distributed within the macropores, effectively filling mesopores.

A macropore‐coordinated graphite‐silicon composite was proposed by manipulating pore distribution via carbon‐blocking.^[^ ^31^ ^]^ The lithiation–delithiation process of Si‐blocking in mesopores at the single‐particle level was simulated using the finite element method (FEM), as shown in Figure 9d. In this design, graphite effectively provided the necessary stress to limit the expansion of Si‐blocking within the macropores during lithiation, thus ensuring the overall stability of the electrode. This unique hybrid anode structure minimized electrode swelling, achieving a high ICE of 93% in half‐cell testing under industrial conditions. Consequently, the MGS anode exhibited a higher volumetric capacity of 493.9 mAh cm⁻^3^ compared to the GS anode (367.5 mAh cm⁻^3^) after 100 cycles, as illustrated in Figure 9e.

Finally, full‐cell configuration demonstrated a higher energy density (1825.7 Wh L^−1^) than the conventional graphite (361.4 mA h cm^−3^ and 1376.3 Wh L^−1^) after 100 cycles. Furthermore, to further enhance the interface stability and suppress the reaction between Si particles and HF, zinc oxide (ZnO) has been explored as a modified coating layer. This ZnO coating improves the compatibility between the electrolyte and active materials.^[^ ^107^ ^]^ Huang et al.^[^ ^108^ ^]^ designed core–shell structured Si‐graphite hybrids through a liquid phase self‐assembly synthesis and annealing treatment. In this approach, graphite particles were coated with ZnO‐incorporated and carbon‐coated Si denoted as Gr@ZnO‐Si‐C composites. Consequently, the designed Gr@ZnO‐Si‐C composites delivered a high reversible capacity of 780 mA h g^−1^ after 1000 cycles at 1.2 A g^−1^.

In order to meet industrial standards, Si nanolayers embedded in graphite/carbon hybrids play a vital role in maximizing energy density while maintaining structural integrity during cycling. However, the manufacturing process must ensure uniform dispersion and precise control of the Si nanolayers to prevent strain‐induced degradation. In addition, the graphite/carbon framework must be optimized to not only buffer the volume expansion but also promote the effective diffusion of Li^+^ and maintain the conductivity of the entire electrode. Finally, the Si nanolayer needs to be protected by an artificial SEI film, and achieving uniform double‐layer coating on the surface of the conductive skeleton is a major challenge.

### 2.3 Modification of Microscale Silicon Composites

#### 2.3.1 Modification of Microscale Bulk Porous Silicon Composites

To achieve higher energy density in LIBs, it is essential to replace low‐specific‐capacity anode materials with those exhibiting higher specific capacities whenever feasible. MSBMs typically exhibit a lower specific surface area compared to their nanoscale counterparts, which can be advantageous for enhancing the ICE. In addition, the desired processing cost and higher packed tap density of MSBM electrodes strongly favor their industrial application.^[^ ^37^, ^109^, ^110^ ^]^ However, addressing the above limitations requires the rational design of SBMs with a high tap density, minimal electrode thickness swelling, and large areal capacity (targeting >3.0 mAh cm⁻^2^ for commercial LIBs).^[^ ^32^ ^]^ Achieving these objectives, alongside a cost‐effective and scalable preparation method, remains both highly desirable and technically challenging.

To enhance the electrochemical performance of Si MPs, it is crucial to apply a high‐quality carbon coating and provide adequate buffer space to accommodate the volume expansion during cycling. To address these challenges, a facile synthesis method was developed to encapsulate Si MPs with highly conformal graphene cages using electroless nickel (Ni) deposition followed by carburization.^[^ ^46^ ^]^ The Ni layer serves a dual purpose: it acts as a template for the growth of graphene, ensuring complete and conformal coverage, while also creating a tunable void space that allows for the expansion of Si during lithiation. The resulting graphene cage structure enables Si MPs to expand and fracture without compromising the stability of the electrode, forming a robust SEI. As a result, the graphene‐caged Si MPs exhibited improved SEI stability and superior cycling performance, retaining 90% of their capacity after 100 cycles.

**Figure 10:**
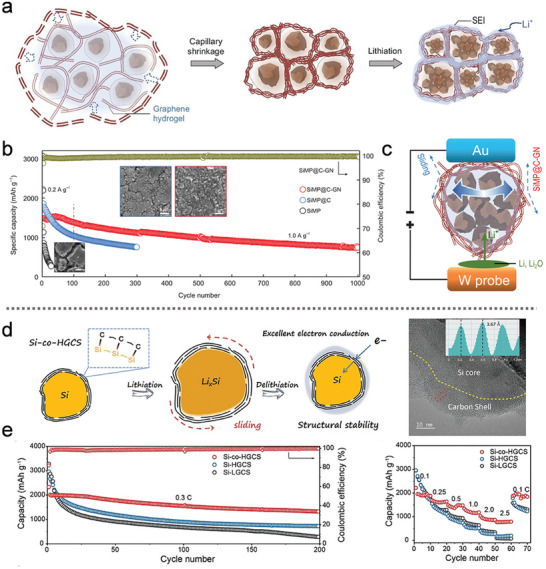
a) Schematic illustration of the lithium‐ion insertion and extraction behavior in the SiMP@C‐GN composite. b) Half‐cell discharge capacities of SiMP, SiMP@C, and SiMP@C‐GN anodes at 1.0 A g⁻¹, along with the corresponding morphology changes (Inset: SEM images). c) In situ lithiation of the SiMP@C‐GN composite observed through a micro‐cell setup. (a–c) Reproduced with permission.^[^ ^111^ ^]^ Copyright 2019, Oxford University Press on behalf of China Science Publishing & Media Ltd. d) Schematic illustration of the lithium‐ion insertion and extraction behavior in the Si‐co‐HGCS composite and its HRTEM images. e) Cycling performance and rate capability of Si‐co‐HGCS, Si‐HGCS, and Si‐LGCS composites. (d,e) Reproduced with permission.^[^ ^112^ ^]^ Copyright 2023, Wiley‐VCH.

However, the use of Ni as a catalyst for graphene growth presents challenges, as the dissolution and precipitation of Ni can lead to difficulties in controlling the uniformity and quality of the graphene coating. To overcome this, a novel carbon capsule cellular (3C) architecture was designed, inspired by plant cell structures, to accommodate the volume expansion and fracture of Si MPs during cycling, as depicted in **Figure** **10**a.^[^ ^111^ ^]^ The Si MPs were coated with a CVD carbon layer, forming a crumpled, densified graphene network. This design effectively balances strength and ductility, allowing for better accommodation of volume changes during cycling. The resulting SiMP@C‐GN composite exhibited superior cycle performance, maintaining stable capacity at 1.0 A g⁻¹ compared to electrodes without buffer gaps or structural support (Figure 10b).

To further evaluate the structural integrity, in situ electrochemical electron microscopy was employed, as shown in Figure 10c. The SiMP@C‐GN anode demonstrated “imperfection‐tolerance” to the volume changes of irregular Si MPs, maintaining electrode integrity over 1000 cycles. Additionally, when incorporated into a SiMP@C‐GN/NCM811 pouch full‐cell, the composite achieved an impressive volumetric energy density of 1048 Wh L^−1^ and an area capacity of 2 mA h cm^−2^. Despite these advancements, the state‐of‐the‐art carbon coating still faces challenges in ensuring stable physical and electrical contact between the carbon shell and the Si MPs, particularly due to the significant size changes that occur during cycling.

In response to the challenges of maintaining a stable Si‐carbon interface, a flexible covalent coating structure was developed to establish a dynamic connection between the Si MPs and the sliding graphene layers, as shown in Figure 10d.^[^ ^112^ ^]^ The covalent bond formed between the Si and the carbon shell ensured a persistent electrical connection and structural reversibility, effectively suppressing deformation during cycling. The structure of the Si‐co‐HGCS (Si composites with high graphite carbon shell and covalent bonding) composite with this covalent linkage was further confirmed through high‐resolution transmission electron microscopy (HRTEM) imaging. As a result, the Si‐co‐HGCS anode demonstrated optimal performance, with the higher graphitic carbon shell maintaining stable bonding between Si and carbon throughout volume expansion, as depicted in Figure 10e. Additionally, the mechanically flexible and complete carbon coating helped mitigate side reactions and buffer the stresses induced by volume expansion. This resulted in significantly improved cycling stability for the Si‐co‐HGCS//NCM811 full cell, which achieved a reversible areal capacity of 3 mAh cm^−2^ with a mass load of 3.05 mg cm^−2^ over 100 cycles.

Compared to Si MPs, SiO_x_ MPs offer the advantages of smaller volume expansion and lower cost, making them more favorable for commercialization. However, SiO_x_ MPs typically have a higher oxygen content, which results in lower electronic conductivity and the formation of a large number of irreversible products during cycling.^[^ ^42^, ^113^ ^]^ To address these challenges, Liu et al.^[^ ^47^ ^]^ developed vertical graphene‐encapsulated SiO microparticles (denoted d‐SiO@vG) through a simple and scalable CVD process. The unique vertical graphene coating created a stable conductive network and provided ample channels for lithium‐ion transport. As a result, the d‐SiO@vG anode exhibited excellent cycling stability, retaining 93% of its capacity after 100 cycles. Additionally, Si nanodomains embedded within a porous SiOx matrix were coated with thin carbon layers using a one‐pot spray pyrolysis method.^[^ ^114^ ^]^ In the precursor solution, NaOH was used to etch the Si nanoparticles, controlling both the size of the Si domains and the Si‐to‐O ratio. Citric acid was then employed as a carbon source to terminate the etching process. The resulting Si‐based active material demonstrated strong electrochemical performance, with an ICE of 80.2% and 87.9% capacity retention after 100 cycles at a 1 C rate.

The well‐coated graphene layers play a crucial role in enhancing electronic conductivity and inhibiting irreversible reactions between the electrolyte and SiO particles. To achieve this, a straightforward approach was used to in situ synthesize a graphene‐coated disproportionated SiO (D‐SiO@G) anode by employing low‐cost coal‐derived humic acid (HA) as the carbon source.^[^ ^115^ ^]^ The D‐SiO@G anode exhibited an initial discharge‐specific capacity of 1937.6 mA h g^−1^ at 0.1 A g^−1^, along with an ICE of 78.2%.

Additionally, a dual‐shell coating structure denoted as SiO_x_@TiO₂@C was developed. This composite consisted of SiO_x_, anatase‐phase TiO₂, and a highly graphitized carbon layer.^[^ ^116^ ^]^ Compared to a pure carbon layer, the anatase TiO₂ provided more effective and rapid Li^+^ ion diffusion in the SiO_x_ material due to the rapid insertion/extraction of Li⁺ along the TiO₂ (001) surface. Furthermore, TiO₂ synergistically elongates the Si‐O bond around the Ti atom, making the Si‐O bond easier to reduce carbothermally. X‐ray photoelectron spectroscopy (XPS) results show that the incorporation of TiO₂ facilitates the carbothermal reduction of SiO_x_. The oxygen content in the SiO_x_@TiO₂@C sample was 1.13, compared to 1.31 in the SiO_x_@C sample, indicating a more efficient reduction process. The SiO_x_@TiO₂@C electrode also demonstrated a higher Li⁺ diffusion coefficient (8.20 × 10^−14^ cm^2^ s^−1^) compared to the SiO_x_@C electrode (3.86 × 10^−14^ cm^2^ s^−1^), highlighting the positive effect of TiO₂ nanocrystals on electrochemical performance. As a result, the SiOx@TiO₂@C anode, with its multifunctional dual‐shell layer, provided numerous rapid diffusion channels for both electrons and Li⁺, while also restraining internal volume expansion to maintain electrode integrity. And, the TiO₂ layer helped suppress side reactions with the electrolyte, contributing to an improved ICE of 81.2%. This anode demonstrated stable cyclability, with 89.5% capacity retention after 800 cycles at 1 A g^−1^, and reinforced rate capability (949.7 mA h g^−1^ at 10 A g^−1^).

In another development, the introduction of magnesium through a vacuum evaporation process allowed for the in situ formation of magnesium‐doped (SiMg_y_O_x_) microparticles, which were blended with a carbon layer and commercial graphite to form the anode (denoted as G‐SiMg_y_O_x_@C).^[^ ^117^ ^]^ The structural design of SiMg_y_O_x_ focused on the homogeneous distribution of inactive magnesium silicate, which helped alleviate volume variations and provide a strong bonding network to prevent particle pulverization. When this G‐SiMg_y_O_x_@C anode was paired with a commercial LiNi_0.8_Co_0.15_Al_0.05_O_2_ (NCA) cathode in a 21700 cylindrical battery, the battery maintained 80% of its initial capacity after 1000 cycles under industry‐recognized testing conditions.

Nevertheless, the coated porous carbon is often ineffective at preventing electrolyte infiltration, and the rigid carbon shells are prone to fracture during repeated volume changes.^[^ ^118^ ^]^ Therefore, adopting a flexible coating strategy is crucial to maintaining the dynamic structural integrity of Si‐based anodes. In this regard, a conductive polymer, polyparaphenylene (PPP), was employed to form a flexible surface coating, ensuring complete shielding of the Si surfaces.^[^ ^119^ ^]^ A core–shell‐structured Si/PPP composite was synthesized via a simple mechanochemical method. During this process, PPP molecules formed a strong plane orientation on the electron‐rich Si surface, facilitated by the polymer's delocalized π‐electron conjugated structure. Moreover, as an n‐type redox‐active polymer, PPP exhibited strong elasticity, which enabled reversible Li^+^ transport. Consequently, the outer PPP layer not only preserved the structural stability of the electrode but also prevented electrolyte infiltration into the Si core. As a result, the Si/PPP composite exhibited stable cycling performance, achieving a capacity of ≈2387 mA h g^−1^ at 0.1 C after 500 cycles with 88.5% capacity retention.

Additionally, covalent‐organic frameworks (COFs) have been investigated as artificial SEIs to enhance the electrochemical performance of SiNPs.^[^ ^120^ ^]^ As two‐ or 3D polymers, COFs offer significant advantages for electrochemical applications due to their high porosity, tunable pore structures, and excellent electrochemical stability.^[^ ^121^ ^]^ The ion‐conducting COFs feature an ordered pore network and modified chemistry, which provide a directional pathway for lithium‐ion migration. Specifically, lithium‐conducting COFs have been used as artificial SEIs to improve the electrochemical performance of Si NPs by facilitating lithium‐ion transport while blocking electron flow.^[^ ^120^ ^]^ As a result, the Si@COF NPs electrode demonstrated significantly improved cycling stability, with the COFs enhancing both lithium‐ion transport kinetics and the mechanical properties of the Si electrode.

**Figure 11:**
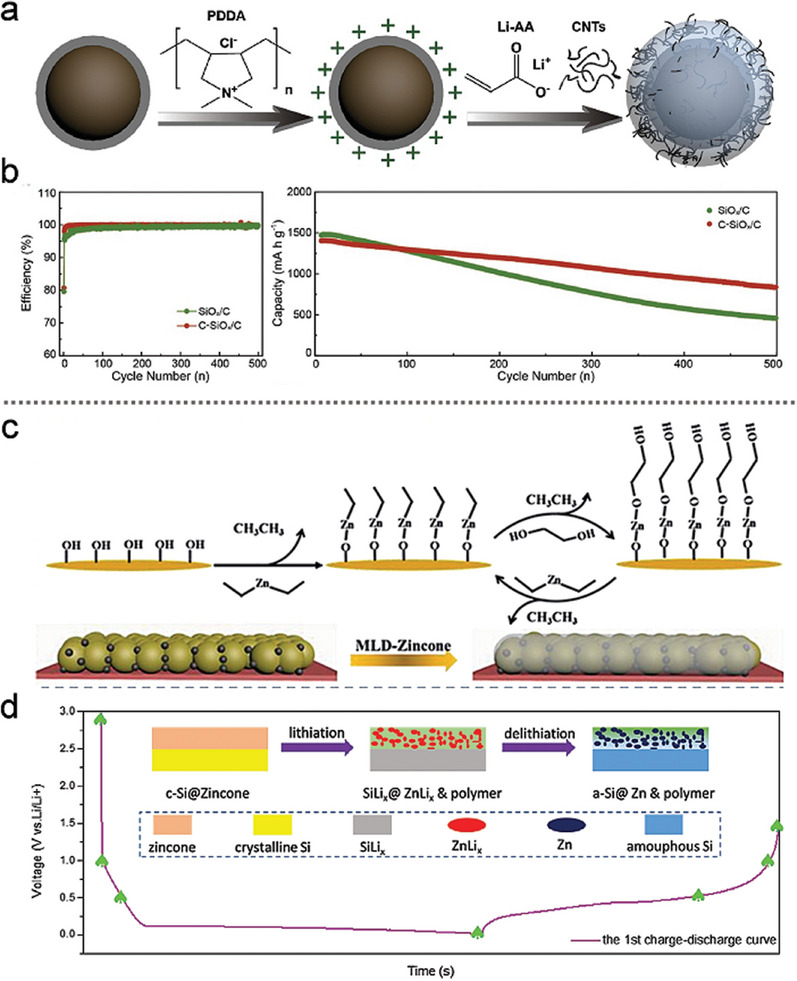
a) The preparation process of micron‐sized C‐SiO_x_/C particles, and b) Coulombic efficiency (CE) and cycling performance of Li||SiO_x_/C and Li||C‐SiO_x_/C electrodes at 0.2 C for the first three cycles and 0.5 C for subsequent cycles. (a,b) Reproduced with permission.^[^ ^110^ ^]^ Copyright 2020, Elsevier Ltd. c) Schematic illustration of the MLD‐zincone‐coated Si in the electrode, and d) the electrochemical evolution of zincone coatings. (c,d) Reproduced with permission.^[^ ^122^ ^]^ Copyright 2021, Wiley‐VCH.

Nevertheless, traditional polymers generally have poor electronic conductivity compared to carbon coatings. To address this limitation, Guo's group^[^ ^110^ ^]^ proposed encapsulating micro SiO_x_ particles in a multifunctional coating, denoted as C‐SiO_x_/C particles, to achieve superior electrochemical performance (**Figure** **11**a). A flexible interface was constructed on the surface of the carbon‐coated SiO_x_ by uniformly incorporating CNTs into the flexible Li polyacrylate (LiPAA) interface. This interface layer, owing to the high stretchability of PAA (582% strain to initial length), maintained the dynamic integrity of SiO_x_ particles during cycling. The combination of superior electronic conductivity and fast ion transport properties enhanced the lithium storage capabilities of the material. In Figure 11b, the development of an ultra‐thin organic polymer film was shown to significantly enhance the electrochemical performance of the active materials while maintaining the overall conductivity of the electrode.

Additionally, Figure 11c illustrates the use of molecular layer deposition (MLD) to precisely apply a zincone polymer coating (≈3 nm) on the Si electrode, thereby creating a stable artificial interface between the anode and electrolyte.^[^ ^122^ ^]^ The electrochemical evolution of the zincone coatings at various potentials was further investigated, providing valuable insights into the mechanisms responsible for the observed improvements in cycling stability and rate capability (Figure 11d). The primary mechanism behind these enhancements is attributed to the ability of the zincone coating and its derivatives to influence the SEI formation pathway, particularly by increasing the LiF content, which helps to stabilize the SEI and mitigate the side reactions commonly observed in Si‐based anodes.

#### 2.3.2 Modification of Microscale Bulk Porous Silicon Composites

MSBMs are particularly vulnerable to fragmentation due to the accumulation of stress from volume changes, which is difficult to mitigate in larger particles. Incorporating buffer pores or spaces within SBMs has thus emerged as an effective strategy to alleviate this stress. Currently, magnesiothermic reduction and chemical etching are widely recognized as simple, rapid, and high‐yield methods for producing mesoporous Si microspheres.^[^ ^123^, ^124^ ^]^ In consideration of raw materials and the complexity of the process, Jia et al.^[^ ^125^ ^]^ developed a cost‐effective approach for preparing micron‐sized porous Si through a combination of microemulsion templating and magnesiothermic reaction. The resulting hierarchical microparticles, which contained uniformly distributed mesopores, were composed of nano‐sized crystalline Si. This hierarchical structure led to a high tap density due to the efficient packing of secondary particles. When 2,3‐dihydroxynaphthalene was used as a carbon precursor to form a thin carbon layer, the resulting porous silicon/carbon (p‐Si/C) material exhibited enhanced structural integrity, preventing fracture and improving electrochemical stability during cycling.

To enhance electronic conductivity and mechanical stability, Zhang's group^[^ ^126^ ^]^ employed polybenzimidazole (PBI) as a novel nitrogen‐enriched carbon source for mesoporous Si microspheres, leading to the formation of a Si‐PBI carbon composite. The resulting Si‐PBI carbon anode demonstrated a high reversible specific capacity (2172 mA h g^−1^), exceptional rate capability (1186 mA h g^−1^ at 5 A g^−1^), and excellent cycling stability. In addition, a scalable and cost‐effective top‐down method was developed to produce porous Si by oxidizing magnesium‐silicon (Mg_2_Si) alloys at low temperatures.^[^ ^127^ ^]^ This approach resulted in a porous Si material with lower oxygen content (8%) compared to commercial Si nanoparticles (12%), and its micro‐sized 3D structure effectively limited electrolyte penetration into the core, reducing the formation of an unstable SEI. As a result, the synthesized porous Si exhibited reduced irreversible lithium loss and superior cycling stability compared to commercial Si NPs. Furthermore, in situ environmental transmission electron microscopy was employed to investigate the reaction mechanism during the controlled oxidation of Mg_2_Si, revealing the formation of MgO and Si.

For the rational design of bulk nanoporous structures, a carbon‐coated ant‐nest‐like microscale porous Si (AMPSi@C) was synthesized by thermally nitriding magnesium‐silicon (MgSi) alloy in nitrogen, followed by chemical etching and coating with a dopamine‐derivative carbon layer.^[^ ^128^ ^]^ The resulting 3D interconnected silicon nanoligaments, with widths of ≈10 nm and bicontinuous nanopores in the AMPSi structure, allowed the electrode to experience negligible outward expansion at the particle level during cycling. The space‐efficient packing of microscale AMPSi contributed to a high tap density of 0.84 g cm^−3^ and a small surface area of 12.6 m^2^ g^−1^. The AMPSi@C anode demonstrated a high ICE of 80.3% and a large volumetric capacity of 1712 mA h cm^−3^ at 420 mA g^−1^. When paired with aLi(Ni_1/3_Co_1/3_Mn_1/3_)O_2_ (NCM111) cathode, the prelithiated AMPSi@C anode enabled the full‐cell configuration to achieve a high energy density of 502 Wh kg^−1^, with excellent capacity retention of 84% after 400 cycles.

**Figure 12:**
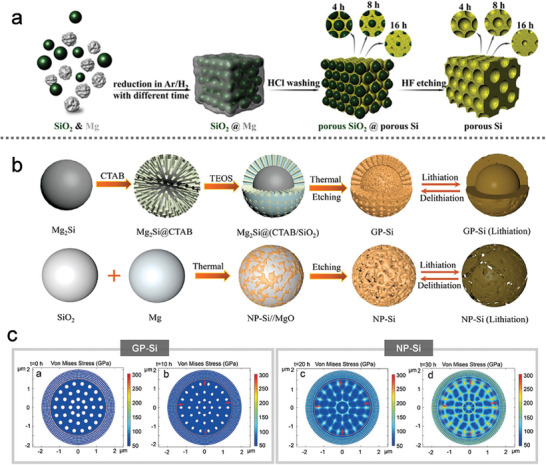
a) Porous Si synthesis process with different etching times. (a) Reproduced with permission.^[^ ^129^ ^]^ Copyright 2020, American Chemical Society. b) The illustration for the fabrication process of GP‐Si and NP‐Si, and c) their Von Mises Stress distribution and volume change before/after discharge. (b,c) Reproduced with permission.^[^ ^131^ ^]^ Copyright 2022, Wiley‐VCH.

Xia's group^[^ ^129^ ^]^ demonstrated that mesopores and larger pores positively influence the reduction of volume expansion, while the presence of micropores is more favorable for lithium‐ion storage. As shown in **Figure** **12**a, a 3D hierarchical macro‐/mesoporous Si structure was synthesized via magnesiothermic reduction. By adjusting the reduction time and post‐treatment processes, the pore diameter, porosity, and wall thickness of the 3D network were systematically tuned. The combination of both macro‐ and meso‐sized pores allowed the Si anode to accommodate significant volume changes, provided ample channels for electrolyte infiltration, and ultimately led to improved electrochemical performance. This group also explored the underlying mechanisms that govern the mechanical response during lithiation and its positive effect on the electrochemical performance of macro‐/mesoporous Si anodes. They found that porous Si with thinner walls and larger pore sizes generated less stress at the boundary regions, which helped stabilize the structure and led to enhanced cyclic performance.

To further improve the performance, a controlled carbon coating was applied to the porous Si. A nanoporous silicon@carbon (NPSi@C) composite was fabricated by reacting Mg_2_Si alloy and CO_2_.^[^ ^130^ ^]^ In this unique design, the uniform carbon layer and the rationally engineered porosity of the NPSi@C provided both a conductive network and a buffer for volume changes during cycling. As a result, the NPSi@C anode achieved an impressive cycle life, maintaining 86.3% capacity retention after 2000 cycles at a high rate of 5 A g^−1^.

Despite the effectiveness of magnesiothermic reduction for producing porous Si materials, this process often results in a reduction in framework strength and tap density. To overcome these challenges, a core–shell gradient porous Si (GP‐Si) material was developed by coating a high‐strength core with a porous shell (Figure 12b).^[^ ^131^ ^]^ During the magnesiothermic reduction process (Mg_2_Si+SiO_2_ → 2Si+2MgO), magnesium from the core layer migrated to react with the SiO_2_ shell, leading to the formation of large pores in the high‐strength core. These pores were essential for buffering the substantial stresses caused by volume expansion. After the removal of the by‐product MgO, the porous shell exhibited a uniformly distributed, abundant pore structure, which enhanced the structural stability of the particle surface during cycling. The GP‐Si electrode demonstrated a discharge capacity of 2127 mA h g^−1^ (1489 mA h cm^−3^) after 100 cycles at 1 A g^−1^, significantly outperforming the NP‐Si electrode, which only achieved a discharge capacity of 747 mA h g^−1^ (410 mA h cm^−3^). In Figure 12c, the stress distribution and internal structure of the GP‐Si particle during the first discharge were simulated. As the discharge time extended (with 30 hours representing the fully discharged state), the stress was predominantly concentrated in the core, particularly around the original pore sites. This observation highlights that the rich pore structure of the shell plays a crucial role in redistributing the stress from the core, preventing the crushing of the outer structure, and ensuring the integrity of the electrode.

The introduction of buffer pores in MSBMs effectively increases their specific surface area, but this also leads to excessive consumption of electrolytes and active lithium ions. This results in the rapid deterioration of cycle performance. While conformal carbon coating technologies have been employed to improve Si anodes, the carbon layer often fills the internal spaces, limiting its effectiveness in managing volume expansion and mitigating side reactions at the electrode/electrolyte interface.^[^ ^132^ ^]^ To address these challenges, a nonconformal carbon shell encapsulation approach has been developed, which provides sufficient internal void space to alleviate volume expansion and reduce electrode/electrolyte interfacial side reactions.

In this context, a nonfilling carbon‐coated porous Si microparticle (nC‐pSiMP) was synthesized by coating Si microparticles with a resorcinol‐formaldehyde resin (RF), followed by thermal disproportionation and etching processes.^[^ ^133^ ^]^ This unique Si‐C composite architecture includes ample void space among interconnected Si NPs, allowing for large volumetric expansion during cycling. The nonfilling carbon shell serves to hinder electrolyte penetration into the interior pore structures, which limits SEI formation on the SiMP surface. As a result, the nC‐pSiMPs maintained structural integrity and demonstrated excellent cycling stability, achieving ≈1500 mA h g^−1^ at a C/4 rate over 1000 cycles.

In addition, Cui et al.^[^ ^134^ ^]^ fabricated densely packed micrometer‐sized secondary clusters through a mechanical approach that combined high pressure, thermal sintering, and high‐energy mechanical milling (HEMM). This process resulted in a high tap density and low surface area nanostructured Si anode, which showed improved electrochemical performance. Notably, the calcined pellets bridged neighboring NPs, which was crucial for the mechanical stability of the clusters during subsequent processes. The addition of the CNT secondary clusters network further enhanced the rate capability by providing an efficient electron transfer pathway. This approach introduced a new methodology for producing low‐cost, high‐energy‐density LIBs with high yield and throughput.

The yolk–shell structure of the Si@graphene cage composite was prepared by the spraying process, magnesiothermic process, and CVD process.^[^ ^135^ ^]^ Mesoporous silica spheres were first reduced to Si, leaving by‐products such as MgO, Mg_2_Si, and unreacted Mg. Nitrogen‐doped graphene cages were then deposited using these by‐products as catalysts and acetonitrile vapor as both the carbon and nitrogen sources. After acid etching to remove the by‐products, mesoporous Si and an empty space within the composite were obtained. This yolk–shell structure of graphene@Si exhibited a high specific capacity and excellent cycling performance, making it a promising candidate for next‐generation Si‐based anodes.

**Figure 13:**
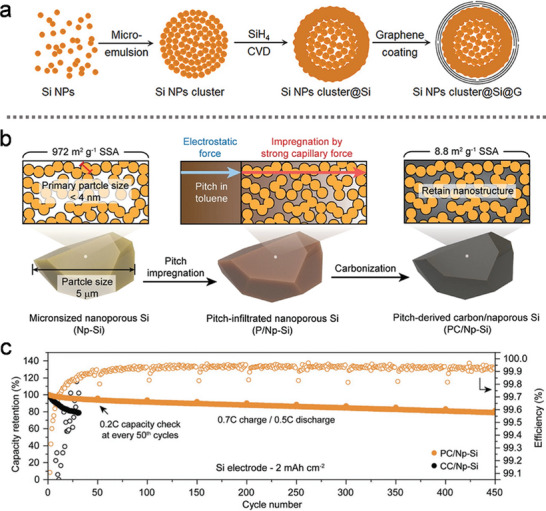
a) The Scheme of the fabrication process of Si NPs cluster@Si@G composite. (a) Reproduced with permission.^[^ ^136^ ^]^ Copyright 2018, American Chemical Society. b) Schematic illustration of the fabrication process of PC/Np‐Si particles. c) Cycling performance of PC/Np‐Si and CC/Np‐Si in the full cell paired with an NMC532 cathode. (b,c) Reproduced with permission.^[^ ^138^ ^]^ Copyright 2021, Wiley‐VCH.

Most nanostructure designs for Si anodes incorporate void spaces to accommodate the significant volume changes that occur during cycling. However, their mechanical stability is often insufficient to withstand the high pressures experienced during the electrode fabrication process, leading to structural failure in some cases.^[^ ^21^, ^39^, ^64^ ^]^ In **Figure** **13**a, Cui's group^[^ ^136^ ^]^ developed a pressure‐resistant Si structure by designing a dense Si shell to coat Si NP clusters, followed by CVD growth of graphene. This composite, referred to as Si NP cluster@Si@G, demonstrated superior electrochemical stability. The excellent cycling performance of this composite can be attributed to two key features: First, the Si skin acts as a strong, protective shell, significantly enhancing the mechanical stability of the anode and enabling it to withstand high pressures (over 100 MPa). Second, the graphene layer, serving as a flexible buffer, and the internal void space within the Si core, together accommodate the volume expansion during cycling. This design not only prevents mechanical failure but also limits the surface area exposed to the electrolyte, thereby improving the ICE.

Subsequently, a surface‐engineered approach was developed to encapsulate mesoporous Si (Mp‐Si) microparticles with a dense Si skin and a conformal graphene cage.^[^ ^137^ ^]^ This hierarchical Si microstructure design effectively minimized direct contact with the electrolyte and internal components, thereby reducing SEI formation. As a result, the encapsulated Mp‐Si@Si@G anode exhibited significantly enhanced CE during both the initial and subsequent cycles. Additionally, the combination of the graphene cage and mesoporous structure provided a buffering effect to accommodate the volume expansion of Si, thus maintaining structural integrity and ensuring excellent cycling stability.

To prevent carbon filling from hindering electrolyte infiltration and to enhance 3D electronic conductivity, a micrometer‐sized Si/C composite with tailored porosity was developed by impregnating petroleum pitch into micrometer‐sized nanoporous Si (denoted Np‐Si) under vacuum, followed by a carbonization process (Figure 13b).^[^ ^138^ ^]^

During the infiltration process, electrostatic interactions between the toluene‐dissolved pitch and Si improved the absorption affinity, ensuring a homogeneous coating. After carbonization, a complete pitch‐derived carbon layer was formed, which minimized electrolyte penetration into the Np‐Si core. The preserved porous structure within the composite effectively accommodated the large volume expansion of Si during cycling, providing numerous electronic pathways while limiting the growth of the SEI film on the surface. Moreover, the core region of the Si particles maintained its original morphology after 450 cycles due to minimal swelling at both the particle and electrode levels. As a result, the micrometer‐sized nanoporous Si, protected by pitch‐derived carbon (denoted PC/Np‐Si), exhibited 90% capacity retention after 50 cycles. In Figure 13c, full cells using PC/Np‐Si as the anode and NMC532 as the cathode demonstrated 80% capacity retention after 450 cycles. Furthermore, a dual carbon‐hybridized hierarchical composite, MSS@CC@CS, was also fabricated, which combined a conformal carbon coating with a nonfilling carbon shell encapsulation.^[^ ^139^ ^]^ The MSS@CC@CS anode exhibited low electrode swelling, an improved ICE of 90.5% at 0.5 C, and exceptional cycling stability, with 89.8% capacity retention after 800 cycles.

#### 2.3.3 Modification of Other Microscale Silicon Composites

Si nanowires (Si NWs) and Si nanosheets (Si NSs) provide several advantages, such as extending the lithium‐ion diffusion path in SBMs and increasing the areal capacity of electrodes at a given thickness.^[^ ^140^, ^141^ ^]^ Using micron‐sized secondary particles assembled from Si NWs or Si NSs represents a promising design strategy with potential for industrial applications.^[^ ^141^, ^142^, ^143^, ^144^, ^145^, ^146^ ^]^ This approach allows for improved electrochemical performance while retaining the structural integrity of the electrode. However, despite these advantages, the design and fabrication of micron‐sized secondary particles assembled from Si NWs or Si NSs face significant challenges. One of the key obstacles is the complexity of synthesizing these structures at a scale suitable for large‐scale production. Additionally, cost considerations and the lack of standardized testing protocols hinder their widespread adoption. As a result, current applications typically involve adding Si NWs or Si NSs in small quantities as additives to enhance the performance of the anode material, rather than as a primary constituent.

Inspired by the exceptional elasticity of wool balls, a multiscale buffering concept was proposed to fabricate micro‐sized, wool‐ball‐like Si‐C materials using micro‐Si as a precursor.^[^ ^147^ ^]^ This design leverages the resilience of wool ball structures to create Si‐C anodes capable of buffering volume expansion. The fabrication was achieved through a radio frequency induction thermal plasma system combined with a spray‐drying procedure, enabling industrial‐scale production at a rate of ≈300 g h^−1^. The resulting wool‐ball‐like Si‐C anodes exhibited high ICE (≈90%), enhanced cycling stability (2000 mA h g^−1^ for 1000 cycles at 4.2 A g^−1^), and superior rate capability (625 mA h g^−1^ at 12.6 A g^−1^). These results demonstrated that the Si/C nanowire‐based wool‐ball framework provided a robust multiscale elastic buffering design, effectively maintaining electrode stability while facilitating rapid Li⁺ and electron transport.

**Figure 14:**
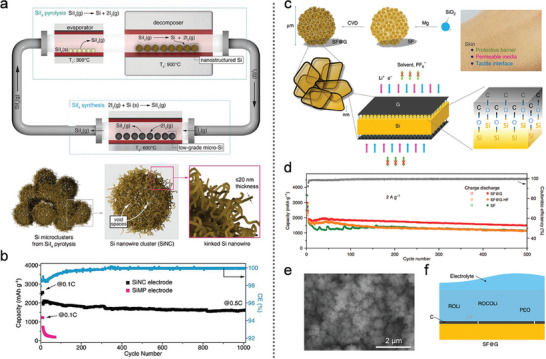
a) The scheme of the synthetic process of secondary particle clusters of Si NWs, and b) the corresponding cycle performance of the SiNC electrode (black) and SiMP electrode. (a,b) Reproduced with permission.^[^ ^148^ ^]^ Copyright 2020, Wiley‐VCH. c) The manufacturing process of two‐dimensional covalently bound Si‐C hybrid materials (namely, SF@G) and the functions of the “skin‐formation”, and d) cycling performance of the SF@G and control electrodes at 0.2 A g^−1^. e) SEM images of SF@G after 100 cycles and f) Schematic description of the interface of cycled SF@G. (c–f) Reproduced with permission.^[^ ^150^ ^]^ Copyright 2020, Nature Publishing Group.

As shown in **Figure** **14**a, a cost‐effective synthesis procedure has been proposed to generate nearly ideal secondary particle clusters of Si NWs.^[^ ^148^ ^]^ A key innovation in this method is the in situ generation of Si iodide (SiI₄) from iodine gas and low‐grade Si microparticles. The decomposition of SiI₄ into crystalline Si and iodine gas not only provides a sustainable method for producing high‐quality Si NW clusters but also enables the recycling of iodine for subsequent cycles. The cyclic iodide process, which involves the formation and pyrolysis of SiI₄, results in Si clusters with kinked nanowires, exhibiting ample void spaces between each nanowire and demonstrating 83.6% capacity retention over 1000 cycles (Figure 14b).

To further increase the energy density and achieve higher tap densities, Si NWs grown on graphite composites (denoted Gt‐Si NWs) were synthesized via a one‐pot scalable approach. To increase energy density and attain tap densities, Si NWs grown on a graphite composite (Gt‐Si NWs) were prepared via a one‐pot scalable approach.^[^ ^149^ ^]^ The uniform distribution of Si NWs on the graphite surface accommodated the large volume changes that occur during cycling, ensuring the mechanical and electrical integrity of the electrode. Additionally, a thin native organic shell grown from the organosilane precursor effectively prevented the oxidation of Si NWs, preserving their structural integrity in air. The resulting Gt‐Si NWs, comprising 32 wt.% Si, exhibited capacity retention of 87% after 250 cycles at a 2C rate, further demonstrating the promising potential of this hybrid material for high‐performance applications.

In addition to secondary particle clusters of Si NWs, silicene flowers (SF), assembled from two‐dimensional Si nanosheets (NSs), have also attracted significant attention in the development of advanced anode materials. SF can be synthesized from industrial byproducts, such as silica fume, via magnesiothermic reduction.^[^ ^140^ ^]^ The resulting SF material is characterized by inherently interconnected nanoplates, which not only accommodate the volume changes during cycling but also provide robust 3D pathways for both electron and Li⁺ transport. This structure ensures both structural and interfacial stability. The SF anode, with a high tap density, exhibited excellent cycling stability (1100 mA h g^−1^ at 2000 mA g^−1^ over 600 cycles) and impressive rate capability (950 mA h g^−1^ at 8 A g^−1^), demonstrating the potential of SF‐based materials for high‐performance applications.

Recently, a scalable and simple manufacturing process was developed by Zhi et al., without compromising the performance parameters of the device.^[^ ^150^ ^]^ They employed a novel two‐dimensional skin‐like covalent encapsulation strategy to mitigate the large volume change experienced by Si electrodes during cycling, as illustrated in Figure 14c. The “skin‐formation” process involved the partial reduction of a silicon oxide layer on Si nanoplates. At high temperatures, the introduction of hydrogen gas facilitated the reaction between the silicon oxide and methane carbon, forming Si‐O intermediates, which served as a skin‐like binding interface. This covalent skin not only acted as a protective barrier but also functioned as a permeable medium and an effective interface. The SF@G anode, featuring this unique skin‐like covalent binding, exhibited remarkable improvements in cycling stability, achieving a high‐rate cycling performance of 2 A g^−1^ over 500 cycles (Figure 14d). Further characterization of the cycled SF@G electrode, including interface morphology and chemical composition analysis (Figure 14e,f), revealed the structural origins of the improved electrochemical performance. The two‐dimensional covalent binding in SF@G facilitated stable and rapid electron and ion transport while modifying the interface between the Si core and the electrolyte. This enhanced interface design secured a robust and efficient contact throughout the cycling process, ensuring the long‐term stability and high performance of the SF@G anode.

In the modification process of micron silicon particles, introducing buffer space and conductive coatings with high mechanical properties is an effective way to reduce stress concentration and improve ion transport kinetics. However, pore formation, coating, and the assembly of nanowires or nanosheets present several challenges in large‐scale production. When pore structures are created through chemical etching or gas‐assisted methods, the distribution and morphology of the pores are often uneven. Excessive porosity can reduce the conductivity of the material, thereby affecting the charge and discharge efficiency of the battery. Moreover, uneven pore structures are prone to breaking under stress concentration, leading to changes in pore morphology. Compared to nano‐silicon, micro‐silicon undergoes greater volume expansion and mechanical stress during battery cycling, which makes coatings with uneven surfaces, excessive thickness, or poor adhesion more prone to cracking and detachment. In large‐scale production, the fabrication of nanowires and nanosheets is complex and costly, and ensuring the consistency and controllability of assembled micron Si particles becomes a significant challenge. Additionally, the weak bonding between nanowires or nanosheets can result in interface failure. Therefore, optimizing the pore structure, coating process, and the stability and uniformity of nanostructures is key to achieving large‐scale production and ensuring the long‐term stability of micron Si‐based composites.

## 3 Battery System Optimizations

### 3.1 Silicon‐based Anode Modified Based on Liquid Electrolyte

In recent years, significant efforts have been focused on optimizing and modifying the surface/interfaces of silicon‐based composites for enhanced performance in energy storage applications.^[^ ^26^, ^151^ ^]^ A critical area of research has been understanding the complex relationship between the mechanical properties of the SEI and the composition of the electrolyte.^[^ ^152^, ^153^, ^154^ ^]^ In many cases, the insulating SEI film acts as a passivation layer, which stabilizes the solid‐liquid interface between the silicon‐based anode and the electrolyte. This stabilization facilitates the transport of Li⁺ while preventing the further decomposition of the solvent and salt, thereby improving the overall efficiency of the battery.^[^ ^155^, ^156^, ^157^ ^]^ Regrettably, the continuous breakage and reformation of the SEI film, induced by the repeated volume expansion and contraction of the anode during cycling, leads to the excessive decomposition of the liquid electrolyte and the consumption of active lithium ions. This degradation accelerates the loss of capacity and undermines the cycling stability of the electrode. To mitigate these challenges, researchers have been exploring various strategies to stabilize the SEI film, reduce electrolyte decomposition, and limit lithium ion loss. Such efforts are essential for improving the overall performance and cycle life of silicon‐based composites in high‐performance energy storage devices.^[^ ^27^, ^28^, ^153^, ^158^ ^]^

**Figure 15:**
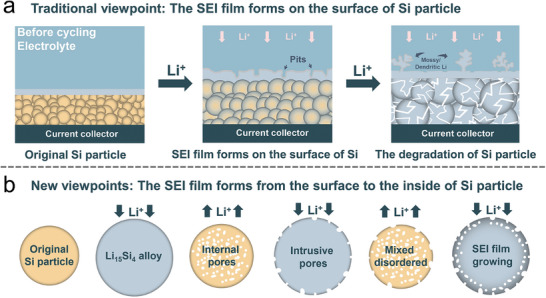
The bidirectional growth of the SEI films: a) Evolution process of electrode structure based on traditional SEI film growth viewpoint. (a) Reproduced with permission.^[^ ^22^ ^]^ Copyright 2012, American Chemical Society; b) The electrode structure evolution process based on the newly discovered SEI film growth viewpoint. (b) Reproduced with permission.^[^ ^28^ ^]^ Copyright 2020, Nature Publishing Group.

In general, researchers identify volume expansion as a primary factor contributing to the failure of silicon‐based electrodes, leading to the continuous growth of the SEI film on the surface of Si. This growth ultimately isolates Si particles and disrupts the conductive network, as shown in **Figure** **15**a.^[^ ^159^ ^]^ Despite significant advancements, even nano‐sized Si has not proven entirely effective in mitigating the issues associated with volume expansion. Recently, Wang et al.^[^ ^28^ ^]^ emphasized that, in addition to volume expansion, vacancies left by lithium ions play a crucial role in Si deactivation, as illustrated in Figure 15b. During cycling, nano‐Si initially exhibits a well‐defined core–shell structure. However, with increasing cycles, irregular pits begin to form on the surface of the nano‐Si particles, leading to the development of interconnected pore channels. Simultaneously, the electrolyte permeates through the SEI film, causing it to grow into the interior of the Si particles. Over successive cycles, the SEI film and fragmented Si particles form a highly mixed and disordered structure. These observations highlight the complexity of addressing poor cycling stability and low CE in Si anodes, which result from the formation of voids, SEI growth, and the associated volume expansion.

It is clear that a single design approach is insufficient to overcome the challenges posed by void formation and volume expansion. A multifaceted strategy is required to address these issues effectively. The design of the material structure, along with the inhibition of electrolyte penetration, emerges as a critical factor in developing Si anode materials that can perform stably in practical applications. Therefore, there is an urgent need to develop a new liquid electrolyte formulation that can produce an ideal SEI film—one that is thin, uniform, mechanically robust, and offers high Li⁺ transport capacity for micro/mesoporous silicon‐based materials. Such an ideal SEI film should not only be able to withstand the continuous swelling and shrinking of the Si anode during cycling but also maintain excellent ionic conductivity and stability throughout long‐term use.^[^ ^159^, ^160^ ^]^

The liquid electrolyte components in LIBs typically include lithium salts, additives, and solvents, which undergo reduction during battery operation to form the SEI film.^[^ ^161^, ^162^ ^]^ Numerous efforts have been focused on enhancing the LiF content in the SEI, as LiF contributes to the film's mechanical stability and ionic conductivity.^[^ ^163^ ^]^ Recently, pentafluorophenyl isocyanate (PFPI) was employed to generate a more resilient and flexible SEI on Si anodes through electron‐induced reductive polymerization.^[^ ^164^ ^]^ The SEI formed in PFPI‐based electrolytes demonstrated greater resistance to the substantial volume changes of Si particles during cycling, effectively passivating the Si surface and reducing active Li^+^ losses. However, the LiF content in the SEI was notably reduced compared to the baseline electrolyte (1 m LiPF_6_ in EC/DEC). In conventional carbonate‐based electrolytes, fluoroethylene carbonate (FEC) has been selectively utilized as an additive to create an SEI with enhanced flexibility and a higher LiF content, which has shown improvements in the cycling performance of Si‐based anodes.^[^ ^165^, ^166^, ^167^ ^]^ Consequently, the composition, structure, and inhomogeneity of the SEI formed with FEC were studied to elucidate the internal mechanisms contributing to performance improvement.

Schlenker et al.^[^ ^166^ ^]^ studied the electrochemical reduction products of EC and FEC, finding that EC reduction was more sensitive to the lithiation state than FEC. First‐principle calculations further supported these findings, demonstrating that the carbon‐fluorine bond in FEC was broken first during lithiation, leading to the formation of a LiF‐rich SEI.^[^ ^168^ ^]^ Yang's group^[^ ^165^ ^]^ observed that the SEI formed on Si/C composite anodes with the FEC system was thicker and denser, which prevented the hydrolysis of LiPF_6_ and hindered the penetration of small molecules into the inner layers during cycling.

**Figure 16:**
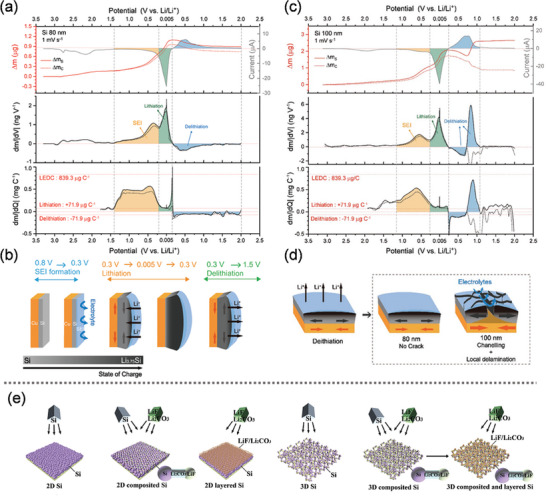
Electrochemical quartz crystal microbalance results and schematics of the cracking behaviors of a,b) the thin‐film and c,d) thick‐film electrodes during the (de)lithiation period. (a–d) Reproduced with permission.^[^ ^169^ ^]^ Copyright 2023, Wiley‐VCH. e) The fabrication process of Si composites by co‐sputtering and sputter‐coating. (e) Reproduced with permission.^[^ ^170^ ^]^ Copyright 2023, Wiley‐VCH.

While electrolytes with FEC have made notable progress, further improvements are still needed. Yoon et al.^[^ ^169^ ^]^ investigated the role of the SEI using electrochemical quartz crystal microbalance and scanning electron microscope studies to better understand and guide its design. **Figure** **16**a,c illustrates the real‐time volume change and stress development of thin‐film and thick‐film electrodes. When tensile stress was below a critical threshold (100–130 nm), the SEI film on the thin‐film electrode remained stable (Figure 16b). However, as tensile stress increased during volume shrinkage, additional SEI formation and interface cracking were observed on the 100 nm thick electrode surface (Figure 16d). Their research confirmed that the FEC‐derived SEI could suppress cracking in thick‐film electrodes and reduce electrolyte decomposition by lowering the stress release rate.

Inspired by the passivation mechanisms observed in stainless steel, Heller et al.^[^ ^170^ ^]^ incorporated mass‐transport blocking layers of LiF/Li_2_CO_3_ into Si anodes through co‐sputtering and sputter‐coating techniques (Figure 16e). This dual‐passivation approach induced the formation of a LiF‐rich SEI, mitigating the volume changes in Si anodes during cycling. As a result, the 3D passivated Si anode exhibited a higher capacity (3701 mAh g^−1^ after 1500 cycles) and better rate capability (up to 50 C) compared to pure Si or Si anodes with single‐layer coatings.

**Figure 17:**
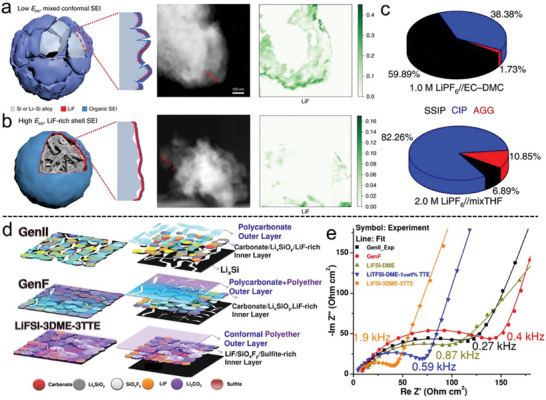
a) Schematic illustration of the cycled Si anodes with an organic, low Eint, and non‐uniform SEI layer, and corresponding HAADF images and EELS spectral images. b) Schematic illustration of the cycled Si anodes with an inorganic, high Ei_nt_, and uniform SEI layer, and corresponding HAADF images and EELS spectral images. c) Distribution of the Li^+^ solvates for the LiPF_6_–mixTHF (2.0 m) and LiPF_6_–EC–DMC (1.0 m) electrolytes from MD simulations. (a–c) Reproduced with permission.^[^ ^171^ ^]^ Copyright 2020, Nature Publishing Group. d) The schemes of the composite (left) and partitioned (right) views of the SEIs formed on the surface of Si particles after cycling. e) EIS plots for various electrolytes at the 100th cycle. The frequency value was taken from the semicircle top for each sample. (d,e) Reproduced with permission.^[^ ^173^ ^]^ Copyright 2021, American Chemical Society.

Exploring the relationship between the mechanical properties of the SEI layer and its corresponding inorganic and organic components is crucial for optimizing the performance of silicon‐based anodes in lithium‐ion batteries. Recent studies have focused on developing novel electrolyte systems designed to improve the cycling performance and ICE of microsized Si anodes.^[^ ^171^ ^]^ A new electrolyte design principle was introduced to achieve this goal by enhancing the mechanical strength and LiF distribution within the SEI film. To investigate the properties of the SEI, advanced characterization techniques such as high‐angle annular dark‐field (HAADF) and electron energy loss spectroscopy (EELS) were employed. The optimized electrolyte, composed of 2.0 m LiPF_6_ in a 1:1 v/v mixture of tetrahydrofuran (THF) and 2‐methyltetrahydrofuran (2‐MeTHF), promoted the formation of a robust LiF‐rich SEI film with low adhesion to the Si anode surface, as shown in **Figure** **17**a,b. This high‐modulus LiF‐rich shell exhibited high interfacial energy (*E*_int_) with the Si anode, which effectively suppressed lithiated alloy pulverization, a common issue in Si anodes. The result was a thinner and more stable SEI film, which contributed to a high ICE of >90%. LiPF_6_ salt plays a key role in the formation of this SEI film, as it can be reduced to LiF at low potentials, avoiding the formation of harmful organic by‐products. By regulating the solvation structure of Li^+^ ions, the aggregation of LiPF_6_ salts in the electrolyte can be improved, leading to the formation of a high‐purity LiF inner SEI layer (Figure 17c). This design ensures that the SEI is thin, insulating, and stable, contributing to the long‐term cycling stability of the anode. As a result, microsized Si anodes in this optimized electrolyte demonstrated a high capacity of 5.6 mAh cm^−2^ over 400 cycles, underscoring the effectiveness of the LiF‐rich SEI in enhancing the electrochemical performance. However, a limitation of this electrolyte system is that it may not be suitable for high‐voltage cathode materials, which could pose challenges for broader applications in high‐energy‐density batteries.

Following this, He et al.^[^ ^172^ ^]^ made significant progress in developing a reinforced and gradient SEI structure on Si NPs to improve the cycling performance and stability of silicon anodes. They created a two‐layer SEI with a buffer layer and a protective layer to accommodate the volume changes inherent in silicon during cycling. This was achieved by first grafting hydrolyzed mercaptopropyl trimethoxysilane (MPTMS), which contains thiol functional groups (‐SH), onto the surface of Si NPs via a condensation reaction. The resulting thiol‐functionalized Si NPs were then heated to 60 °C, initiating a thiol‐ene click reaction between the Si‐SH NPs and vinylene carbonate (VC) in the presence of azodiisobutyronitrile (AIBN). This reaction formed a homogeneous and reinforced SEI that effectively buffered the expansion and contraction of Si NPs during cycling. This design was found to improve the long‐cycle performance of Si‐graphite blending anodes, with enhanced structural integrity during repeated cycling.

In another study, Yang's group^[^ ^173^ ^]^ explored glyme‐based electrolytes (GlyEls) as a means of stabilizing Si anodes in Figure 17d. They investigated the chemistry, structure, and formation mechanism of the SEI in GlyEls and found that these electrolytes helped form a more elastic and Li^+^‐conductive SEI compared to traditional carbonate‐based electrolytes containing 10 wt.% fluoroethylene carbonate (GenF). The GlyEl‐SEI structure exhibited a conformal coating layer with an enriched polyether outer layer and a fluorine‐rich inner layer. This unique structure allowed the SEI to endure stress variations without compromising the silicon anode's structural integrity, keeping it “fracture‐free” throughout cycling. As illustrated in Figure 17e, electrochemical impedance spectroscopy (EIS) plots showed reduced polarization effects and improved cycling performance for the samples using GlyEls. This demonstrates that the elastic and conductive nature of the SEI in GlyEls enhances the overall performance and longevity of the silicon anode by minimizing stress‐induced damage and improving lithium‐ion conductivity.

The incorporation of triethyl phosphate (TEP) as a solvent additive and the development of a nonflammable localized high‐concentration electrolyte (LHCE) represent key advancements in improving the safety and performance of silicon‐based LIBs. Linear carbonate solvents, due to their low flash points, pose significant safety risks, particularly under extreme conditions such as short circuits or overheating. To mitigate these risks, TEP was introduced as a nonflammable additive to the electrolyte, significantly enhancing the safety of silicon anodes in LIBs.^[^ ^174^ ^]^

The LHCE design involves the combination of a dilute solvent, bis(2,2,2‐trifluoroethyl) ether, with a high‐concentration electrolyte that contains 1.2 m LiFSI (lithium bis(fluorosulfonyl)imide) in a mixture of TEP and FEC. The high salt concentration reduces the number of free solvent molecules, leading to the formation of highly coordinated Li^+^‐TEP solvates, which improves the stability of the electrolyte. Moreover, the presence of FEC promotes the formation of a LiF‐rich SEI, which is beneficial for stabilizing the Si anode surface and improving cycling performance. The LHCE design not only enhances the electrolyte's wettability, ensuring better contact with the anode, but it also significantly improves the long‐term stability of silicon anodes. In full‐cell configurations, silicon anodes in this innovative electrolyte showed impressive performance, retaining over 90% of their capacity after 600 cycles at a C/2 rate. This achievement underscores the potential of LHCEs to enhance the cycling stability of Si‐based LIBs while addressing safety concerns associated with conventional electrolytes.

By optimizing the electrolyte composition, the interface failure of the SEI film in the Si anode can be effectively improved. Specifically, the SEI film formed by ether‐based electrolytes demonstrates enhanced flexibility and ionic conductivity. However, the compatibility between the electrolyte and electrode materials remains a significant challenge. The narrow electrochemical window of ether electrolytes limits their compatibility with high‐voltage cathodes, thereby affecting the construction of high‐energy‐density batteries. Additionally, due to the structural differences between Si and graphite, the solvation structure of an electrolyte designed for Si anodes may not be suitable for graphite, further complicating electrolyte optimization. Moreover, the complex composition and structure of the anode materials result in weak adhesion, uneven thickness, and inhomogeneity between the SEI film and the Si surface, making the cycle life of pure Si/C anodes still insufficient to meet commercialization requirements. Therefore, the development of an electrolyte that is compatible with both silicon‐carbon anodes and high‐voltage cathodes is essential for achieving high‐performance, long‐life LIBs.

### 3.2 Silicon‐based Anode Modified Based on Solid‐State Electrolytes

Several strategies have been proposed to address the challenges associated with silicon‐based anode materials, including substantial volume expansion, low electronic conductivity, and poor interfacial stability with liquid electrolytes.^[^ ^175^ ^]^ However, it is crucial to also consider the thermal instability of LIBs when using organic electrolytes, particularly in mobile and grid applications. One promising solution to these thermal stability concerns is the adoption of SSEs. By replacing volatile liquid electrolytes with SSEs, ASSBs incorporating Si anodes can potentially overcome the energy density limitations of conventional LIBs while fundamentally mitigating thermal runaway risks.^[^ ^176^, ^177^ ^]^ Si anodes are considered ideal candidates for ASSBs due to their stability, processability, and cost‐effectiveness when compared to lithium metal anodes.^[^ ^178^ ^]^ However, it is important to note that soft lithium metal can be compressed under pressures exceeding 25 MPa, potentially penetrating the SSE and causing short circuits.^[^ ^179^ ^]^ In contrast, the application of large axial compressive pressures in ASSBs is commonly employed to mitigate the volume expansion of Si, which enhances both the safety and performance of ASSBs with silicon‐based anodes.^[^ ^23^ ^]^

In this context, the influence of external compressive stress on the electrochemical performance of Si‐based anodes, including both commercial Si particles and SSEs, has been extensively studied. Lee et al.^[^ ^180^ ^]^ explored this relationship and demonstrated that elastic SSEs can exert a counteracting force, limiting the volume expansion of Si particles. This behavior is beneficial for maintaining the structural stability of the electrode and enhancing cycle performance. However, it is important to note that excessive external stress can impede the insertion of Li^+^ into Si particles, hindering the Si anode from achieving its theoretical specific capacity.

Carter et al.^[^ ^181^ ^]^ further validated the impact of compressive stress on cell capacity reduction through both analytical and finite element models. They emphasized the role of Young's modulus of the SSE in enhancing electrochemical performance, noting that the high stiffness of the SSE matrix promotes the development of compressive stress. Subsequently, Yamamoto et al.^[^ ^182^ ^]^ observed that optimized compressive stress in practical full‐cell designs facilitated plastic deformation and improved contact between the active materials and the SSE layer, helping to maintain the integrity of the conduction network.

More recently, McDowell et al.^[^ ^183^ ^]^ investigated the relationship between the composite electrode structure and the evolution of compressive pressure (uniaxial stress) in full cells. Their findings indicated that stress variations during charge–discharge cycles were primarily attributed to the large partial molar volume of lithium in the anode materials. These studies underscore the importance of controlling chemo‐mechanical interactions in the composite design of Si particles and SSEs, which is crucial for optimizing the performance of Si‐based anodes.

Currently, significant research has been focused on silicon‐based anodes in ASSBs.^[^ ^176^, ^184^, ^185^, ^186^ ^]^ Based on the interaction between SSEs and active materials, Si anodes can be classified into two main categories: powder electrodes and thin‐film electrodes. For powder electrodes, Si particles, surrounded by SSE particles, create extensive electron and ion conduction pathways throughout the electrode due to the 3D contact area between the active material and surrounding components.^[^ ^178^ ^]^ The electrochemical performance of Si anodes has been significantly improved by incorporating multi‐walled CNTs as conductive additives and carbon volume buffers.^[^ ^187^ ^]^ Subsequently, Lee et al.^[^ ^188^ ^]^ developed a Si‐Sn hybrid anode, where in situ pressure from the lithiation expansion of Sn facilitated the reversible alloying of silicon. Takahashi et al.^[^ ^189^ ^]^ demonstrated that the uniform dispersion of nanoporous Si helped maintain intimate contact with the surrounding SSE, accommodating elastic deformation. Void‐structured composites have also been designed to mitigate the volume changes associated with Si expansion during cycling.^[^ ^190^ ^]^ Additionally, Fang et al.^[^ ^191^ ^]^ proposed an innovative in situ self‐adapting electrochemical grinding (ECG) strategy to prepare stable micron‐sized Si composites. MgH_2_ was used as the grinding aid during the ECG process. As MgH_2_ migrated into cracked Si particles, it gradually transformed into a conductive matrix, comprising electronically conductive Li_x_Mg and ionically conductive components. The internal stresses generated by MgH_2_ effectively accommodated the volume changes of micron‐sized Si particles. The resulting Si anode, prepared via ECG, exhibited excellent capacity retention, maintaining 91% capacity after 200 cycles at 0.5 A g^−1^.

Additionally, a new Si/C composite anode was developed by dispersing carbon‐coated Si particles onto the surface of carbon paper (CP).^[^ ^192^ ^]^ As a 3D conductive substrate, carbon paper facilitated strong integration between Si particles and the polymer electrolyte. The substantial specific surface area of the carbon paper enhanced the adhesion between the different components of the electrode, improving overall structural cohesion. However, it is important to note that this electrode exhibited a relatively low CE of 77%. Further optimization of this composite anode may be required to enhance its CE and improve its performance in energy storage applications.

In contrast to powder electrodes, the SSE in thin‐film electrodes creates a two‐dimensional interfacial plane, rather than a 3D contact network. This configuration promotes good interfacial stability but may compromise ionic conductivity within the electrode. As a result, significant research efforts are being devoted to overcoming the limitations of thin‐film anodes to make them suitable for industrial applications. Yoon et al.^[^ ^193^ ^]^ investigated the effect of carbon content in Si films to enhance their electrochemical performance. They found that introducing a small amount of oxygen into amorphous silicon films via magnetron sputtering improved cycling stability in ASSBs by promoting the formation of inactive nanoarchitecture through conversion reactions.^[^ ^194^ ^]^

**Figure 18:**
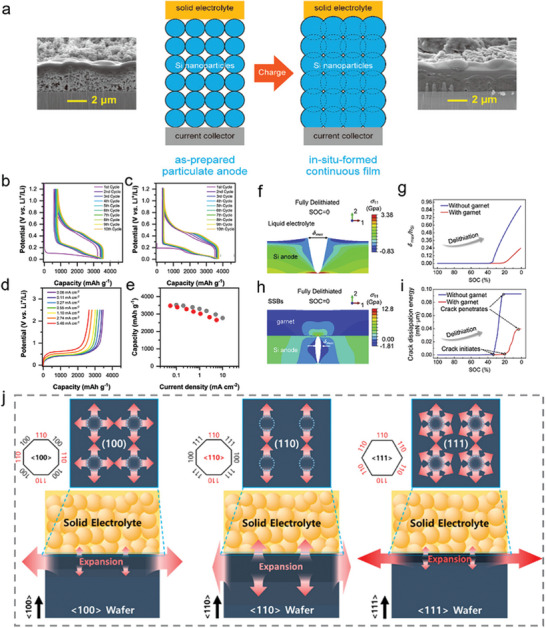
a) The structure evolution of the Si NPs prepared by spray deposition in a solid‐state cell. Galvanostatic charging and discharging potential profiles of the b) spray‐deposited Si particulate anode and c) sputter‐deposited Si continuous film during the first 10 cycles. d) High‐rate discharge capability test results for the Si particulate anode. The constant currents for the color‐coded discharging curves are indicated in the legend. e) Comparison of the high‐rate discharge capability of the spray‐deposited Si particulate anode (red‐filled circles) with that of the sputter‐deposited Si continuous film (gray‐filled circles) as a function of current density. (a–e) Reproduced with permission.^[^ ^195^ ^]^ Copyright 2019, American Chemical Society. (f–i) Mechanical constraints of the garnet‐type solid electrolyte. Finite element modeling of the morphology and normal in‐plane stress distribution of the Si anode in f) liquid batteries and h) ASSBs at the fully delithiated state. g) Comparison of the maximum crack opening displacement *δ*_max_/hSi (normalized by the initial Si anode thickness hSi) without (blue) and with (red) garnet during delithiation. i) Comparison of the crack dissipation energy (mN∙µm) in the Si anode without (blue) and with (red) garnet during delithiation. (f–i) Reproduced with permission.^[^ ^196^ ^]^ Copyright 2019, Elsevier Ltd. j) Cracks evolved along^[^ ^110^ ^]^ direction for each wafer of plane and side views. (j) Reproduced with permission.^[^ ^198^ ^]^ Copyright 2023, American Chemical Society.

In comparison to sputtered Si films, spray deposition was shown to optimize the porous structure of silicon films (**Figure** **18**a).^[^ ^195^ ^]^ The confined space within the spray‐deposited Si films caused volume expansion, which induced the transformation of the film into a continuous structure. This process significantly improved the cycling performance and high‐rate discharge capability of the spray‐deposited Si particulate anode compared to its sputter‐deposited counterpart (Figure 18b–e). However, it is important to note that the nanoscale thickness of these silicon anodes may limit their overall energy density.

To enhance the surface loading of the active material, a solid‐state Si anode with a 1 µm thickness was successfully fabricated using plasma‐enhanced chemical vapor deposition (PECVD).^[^ ^196^ ^]^ The garnet‐type SSE maintained robust solid contact with the Si anode while effectively alleviating its volume expansion, benefiting from its strong nanomechanical constraints. Finite element modeling was used to examine the Li^+^ concentration, stress distribution, and defect formation in the 1 µm Si anode, with the crack propagation process detailed in Figures 18f–i. When the in‐plane tensile stress resulting from the large volume shrinkage exceeded a critical threshold, cracks initiated and propagated along the thickness direction (Figure 18f). The garnet SSE constrained the state of charge due to crack initiation, increasing it from 20% to 36% (Figure 18g). The maximum crack opening displacement (*σ*_max_) in the all‐solid‐state battery was smaller compared to that in a liquid electrolyte battery, as shown in Figure 18h. In comparison to Si anodes without garnet SSE, the nanomechanical constraints provided by the garnet reduced crack dissipation energy, significantly improving the structural durability of the electrode (Figure 18i). As a result, the Si anode demonstrated a higher ICE of 83.2%, compared to 77.1% for Si anodes with organic electrolytes.

Subsequently, columnar silicon (col‐Si) films with a practical areal capacity of 3.5 mA h cm^−2^ were fabricated using a scalable physical vapor deposition process.^[^ ^197^ ^]^ During cycling, the application of external pressure (20 MPa) on the cell stack enabled the col‐Si to exhibit 1D breathing behavior in the vertical direction. The 2D intimate contact between the silicon film and SSE reduced the surface area for side reactions, leading to the formation of a mechanically stabilized lateral SEI. Furthermore, full cells with col‐Si anodes demonstrated enhanced capacity retention of 82% after 100 cycles, along with a high CE of 99.7–99.9%.

Recognizing the challenges associated with composite electrodes made from Si powder—such as the formation of significant internal voids and detrimental interfaces. Lim et al.^[^ ^198^ ^]^ adopted grooved <110> silicon wafers to effectively mitigate crack formation and pulverization. This approach enabled the fabrication of a void‐free, monolithic pure Si electrode. As illustrated in Figure 18j, the grooved <110> silicon wafer exhibited excellent reversible (de)lithiation behavior. The study also explored different wafer orientations, including <100>, <110>, and <111>, demonstrating that the surface grooves significantly increased the interfacial area and promoted conformal contact with sulfide electrolyte layers under the fabrication pressure (≈500 MPa). As a result, the grooved <110> silicon wafer electrode achieved a high areal capacity of 10 mA h cm^−2^ in a half‐cell at ≈25 °C, and 8.8 mA h cm^−2^ in a full‐cell at 60 °C. This innovative design highlights the potential for improving the performance and stability of silicon‐based electrodes in LIBs.

**Figure 19:**
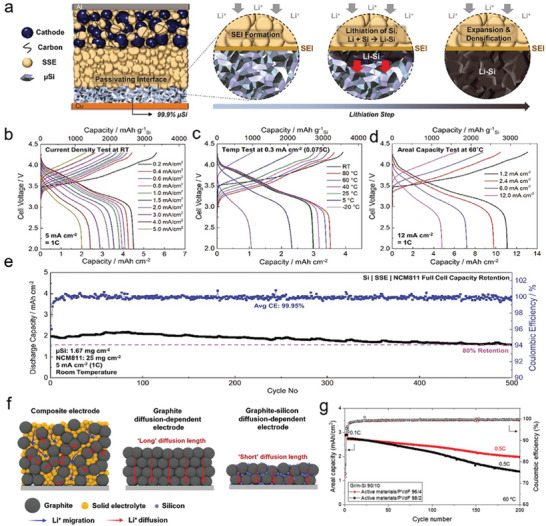
a) Schematic illustration of 99.9 wt.% mSi electrode in an ASSB full cell during lithiation. b–e) µSi||SSE||NCM811 performance: b) High current density test. c) Wide temperature range test. d) High areal capacity test. e) Cycle life at room temperature. (a–e) Reprinted with permission.^[^ ^199^ ^]^ Copyright 2021, American Association for the Advancement of Science. f) Schematic illustration of the structure and lithium‐ion transport paths of the composite electrode, the graphite diffusion‐dependent electrode, and the graphite‐silicon diffusion‐dependent electrode. g) Capacity retention of the graphite–silicon diffusion‐dependent electrode at 60 °C. (f–g) Reproduced with permission.^[^ ^200^ ^]^ Copyright 2022, Wiley‐VCH.

Recent research by Meng et al.^[^ ^199^ ^]^ explored a carbon‐free thin‐film electrode containing 0.1 weight% polyvinylidene fluoride, which effectively mitigates side reactions in low‐cost micron‐sized silicon and sulfide‐based SSEs. This approach significantly improved interfacial stability, as illustrated in **Figure** **19**a. The SSE, which lacks permeability, facilitated the formation of a 2D interfacial plane with the porous thin‐film electrode. Under a uniaxial stack pressure of 50 MPa, tight contact between the micron‐sized Si particles was maintained, ensuring stability within the electron and ion transport network. When assembled into a full cell with the carbon‐free thin‐film anode (containing 99.9 weight% microsilicon) and an NCM811 cathode protected by a boron‐based coating, the cell demonstrated excellent electrochemical performance under high current densities, wide temperature ranges, and high surface capacities (Figure 19b–d). Notably, the full‐cell configuration at 5 mA cm^−2^ exhibited 80% capacity retention after 500 cycles (Figure 19e).

To simultaneously achieve high power and high energy densities in ASSBs, a graphite‐silicon diffusion‐dependent (GSDD) anode was designed to leverage inter‐diffusion between active material particles (Figure 19f).^[^ ^200^ ^]^ During the charge–discharge process, graphite particles supplied sufficient electrons to adjacent silicon, while the uniform distribution of Si NPs shortened the effective diffusion pathway across the entire electrode. Moreover, the graphite particles helped accommodate the volume changes in the silicon NPs. The GSDD anode, containing 10 wt.% silicon NPs, exhibited a capacity retention of 72.7% after 200 cycles at 1.77 mA cm^−2^ (Figure 19g), demonstrating its potential for enhancing the cycling stability of Si‐based anodes in ASSBs. Furthermore, the performance of solid‐state battery materials is influenced by the choice of electrolyte. Han et al.^[^ ^201^ ^]^ found that hydride SSEs not only stabilized the lithium anode but also significantly enhanced the stability of the Si anode. Compared to sulfide‐based electrolytes, hydride SSEs resulted in an unprecedented improvement in the ICE of Si, reaching 96.2%, with the full cell maintaining a stable cycling CE of 99.34%.

To facilitate the commercialization of silicon‐based SSBs, it is essential to identify the key factors that contribute to exceptional electrochemical performance under high areal capacity conditions. In a recent study, thick all‐solid composite electrodes were developed using a combination of Si NPs, a superionic SSE, and a carbon additive.^[^ ^202^ ^]^ The study revealed that high‐rate performance under these demanding conditions could be achieved by expanding the Si/SE contact interface through microstructural optimization, which enhances effective ionic conductivity. At the same time, it was crucial to avoid excessive tortuosity in the well‐distributed composite electrodes. The composite materials, which included small (s‐Si), medium (m‐Si), and large (l‐Si) silicon particles, were analyzed to understand their impact on ionic conductivity. Among these, the m‐Si composite exhibited the highest residual porosity (48.6 vol%) at the same mass fraction. Flux‐based simulations of ionic current distribution indicated that the residual pores contributed to increased ionic tortuosity, which can impede ionic flow.

Compared to lithium metal anodes, ASSBs using Si anodes can overcome the energy density limitations of traditional LIBs, reduce the risk of thermal runaway, and significantly enhance battery safety. Currently, Si‐based ASSBs typically alleviate the volume expansion of silicon anodes by applying axial compressive stress, thereby improving both the safety and cycle performance of the battery. However, excessive compressive stress can impede the insertion of Li^+^ into Si particles, limiting the anode's ability to achieve its theoretical specific capacity. Additionally, in practical applications, it is challenging to maintain a constant, high pressure to ensure the stable operation of the battery. Furthermore, issues such as the contact resistance between the Si anode and the SSEs, the ionic conductivity of thin‐film electrodes, and the stress distribution within the materials remain significant hurdles for the commercialization of ASSB technology. Therefore, further research aimed at addressing the compatibility between silicon anodes and SSEs, limiting volume expansion, and stabilizing the SEI film is crucial for enhancing the safety and energy density of all‐solid‐state batteries.

### 3.3 Silicon‐based Anode Modified Based on Binder

In the pursuit of higher energy densities, enhancing lithium storage capacity by replacing commercial graphite with SBMs is a promising strategy. However, the polymer binders commonly used in commercial batteries are not suitable for adapting to the large and repeated volume changes experienced by silicon anodes. This challenge makes it difficult to maintain strong adhesion between the current collector, active material, and conductive additives.^[^ ^203^, ^204^ ^]^ The issue is especially pronounced for micron‐sized SBMs, where large anisotropic stresses can cause brittle active materials to crack, resulting in rapid capacity degradation.^[^ ^205^ ^]^ Furthermore, traditional binders, which are inactive by nature, fail to provide active sites for electrochemical reactions and hinder electron transport within the electrode. This necessitates the addition of inactive conductive agents, which, in turn, limit the overall energy density of the battery. To address these issues, an ideal binder should possess key attributes, including self‐healing capabilities, excellent stress relaxation properties, enhanced mechanical support for SEI film formation, and high electronic conductivity.^[^ ^206^ ^]^ This makes the binder a potential solution for improving the performance and energy density of silicon‐based material batteries.^[^ ^207^, ^208^, ^209^, ^210^, ^211^ ^]^

**Figure 20:**
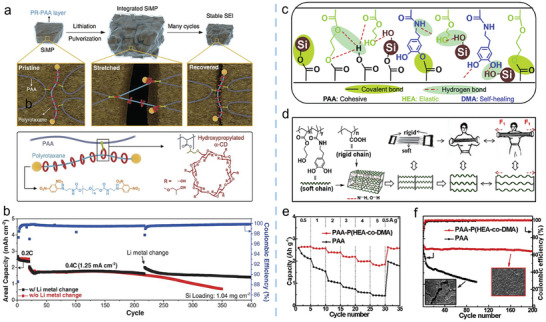
a) Graphical representation of the operation of PR‐PAA binder to dissipate the stress during repeated volume changes of SiMPs, together with chemical structures of polyrotaxane and PAA. b) Discharging capacity retention and Coulombic efficiencies of the PR‐PAA–SiMP electrode when cycled at 0.2 and 0.4 C. 1C = 3.12 mA cm^−2^. Si loading, 1.04 mg cm^−2^. The black arrow indicates the point when the Li metal counter electrode is replaced with a fresh one. (a,b) Reprinted with permission.^[^ ^212^ ^]^ Copyright 2017, American Association for the Advancement of Science. c) Structural formulae of copolymers and their interaction with Si. d) Chemical structures and illustrative interaction of P(HEA‐co‐DMA) and PAA, and the spring expanders model of their complex. e,f) rate performance and cycle performance of the SiMP Electrodes with Different Binders. (c–f) Reproduced with permission.^[^ ^213^ ^]^ Copyright 2018, Elsevier Ltd.

Until 2017, efforts to develop high‐performance polymer binders for MSBM were relatively limited. A significant advancement was made by Choi et al.,^[^ ^212^ ^]^ who introduced a commercially viable electrode with enhanced reversibility by incorporating a stress‐release mechanism based on a pulley action model (**Figure** **20**a). In this system, a sliding‐ring polyrotaxane enabled a highly elastic PAA binder to undergo sliding motion, significantly enhancing the elasticity of the polymer network. The sliding of the polyrotaxanes allowed the modified PAA film (denoted as PR‐PAA) to withstand up to 390% strain without rupture. As a result, the electrode, which utilized PR‐PAA as the binder and featured a silicon loading of 1.04 mg cm^−2^, demonstrated impressive cyclability with ≈1.25 mA cm^−2^ current density, retaining 85% of its capacity after 370 cycles (Figure 20b).

In a different approach, a self‐healing, multi‐network polymer binder was developed by synthesizing poly(acrylic acid)‐poly(2‐hydroxyethyl acrylate‐co‐ dopamine methacrylate) to endure repeated stretching and shrinking without structural degradation (Figure 20c).^[^ ^213^ ^]^ This binder featured a covalently crosslinked rigid body network in combination with abundant hydrogen bonds in the flexible areas (Figure 20d). The multi‐network structure provided mechanical support to the electrode while simultaneously offering self‐healing capabilities, which helped buffer the strain and prevent disintegration of the Si MPs. Further modification involved incorporating polyethylene glycol (PEG) groups into the binder to enhance both its self‐healing ability and lithium‐ion conductivity.^[^ ^213^ ^]^ As shown in Figures 20e,f, the Si MP anode prepared with this self‐healing binder exhibited reasonable rate performance and cycling stability.

Despite significant advancements, the electrochemical performance of silicon‐based anodes has yet to meet the stringent requirements for commercial applications. One of the challenges is that the microenvironment around each individual Si particle undergoes gradual changes until the electrode swelling reaches a saturation point. During lithiation and delithiation, two distinct stages of microenvironmental changes occur. In the initial stage, reversible noncovalent interactions facilitate the adaptability of the binder, allowing it to reposition and reorient following particle shrinkage. However, as the volume change of Si particles becomes more pronounced, these interactions become irreversible, causing permanent shifts in the binder's structure.

To address this challenge, an adaptive polymeric binder conjugated with gallol‐hyaluronic acid (GA‐HA) was developed, inspired by plant‐based phenolic chemistry.^[^ ^214^ ^]^ This binder utilizes noncovalent interactions, such as hydrogen bonds and hydrophobic interactions between the gallol moiety and hyaluronic acid, to provide a strong binding affinity that helps constrain the early stages of volume expansion. As the Si NP volume expands significantly, this triggers a sol‐to‐gel transition, leading to covalent gelation within the binder. This transition creates a stable, flexible, and stretchable microenvironment that better accommodates the large volume changes of Si NPs and helps prevent the formation of morphological cracks. As a result, nano‐sized Si anodes demonstrated enhanced capacity and structural stability, benefiting from the adaptive properties of GA‐HA throughout the cycling process. However, Si Si MPs exhibited only partial improvement, as the large size of Si MPs hindered effective contact between the gallol moieties of the GA‐HA binder, thereby preventing efficient gallol‐to‐gallol covalent self‐crosslinking. This limitation suggests that further optimization is required to enhance the performance of Si MPs with this binder system.

The commercial viability of silicon‐based anodes can be significantly enhanced by designing binders that are tailored to the structure and size of the active material. For example, the PAA binder has been shown to improve the electrochemical performance of modified silicon submicroparticles (SiSMPs) when combined with tannic acid as an active material. The conformal coating of tannic acid facilitates the formation of a 3D cross‐linked network with the PAA binder, which ensures strong adhesion between the battery components, protects the SiSMPs from side reactions with the electrolyte, and maintains the structural integrity of the electrode during cycling.^[^ ^215^ ^]^ In another approach, Hong et al.^[^ ^216^ ^]^ encapsulated Si micron particles entirely by introducing an esterification reaction, which formed a covalent bond between the carboxyl group (–COOH) of the PAA binder and the hydroxyl group (–OH) of graphene oxide (GO). The resulting GO‐PAA composite cage effectively confines the pulverized Si particles during cycling, thereby enhancing the electrode kinetics. This approach led to a Si micron particle electrode that demonstrated stable cycling performance, with a high areal capacity of 1.98 mA h cm^−2^ after 500 cycles.

More recently, Choi et al.^[^ ^217^ ^]^ further advanced Si/C composite anodes by employing a Zn^2+^‐imidazole complex binder, known for its excellent elasticity. The high elasticity of this binder, coupled with the recoverable nature of the Zn^2+^‐imidazole coordination bonds, helps maintain electrode integrity during cycling, while also offering high ionic conductivity. These binder designs highlight the importance of customizing binders to address the specific challenges posed by different sizes and structures of silicon‐based active materials.

An alternative approach to improving electrode performance involves the use of a specially engineered structural conductive agent, which can replace traditional binders and conductive additives, enabling the production of electrodes with a higher proportion of active materials. In this context, a PECVD process was employed to synthesize a network of interconnected carbon‐coated porous silicon nanowires, denoted as N‐PSi@C, where “N” refers to “network” and “P” to “porous”.^[^ ^218^ ^]^ As a binder‐free anode, the N‐PSi@C composite demonstrated excellent rate capability and stable long‐term cycling performance. Several key factors contributed to the enhanced performance of the N‐PSi@C composite. The 3D porous silicon nanowires formed an interconnected network that facilitated efficient electron transport and accommodated the volume changes of the silicon nanowires during cycling. Additionally, the conformal carbon coating provided a continuous electron transport path while mitigating the thickening of the SEI film. This innovative design eliminates the need for traditional binders, thereby allowing a higher proportion of active material in the electrode and enabling a higher electrode loading.

**Figure 21:**
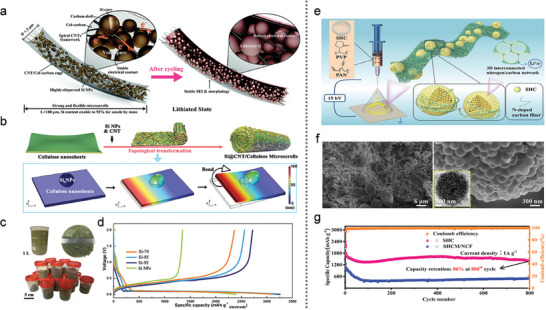
a) one Si@CNT/C‐microscroll before and after electrochemical cycling. b) the schematic illustration of the topological deformation process. c) 1 L of a Si@CNT/cellulose mixed solution along with the amount of Si@CNT/cellulose aerogel produced per batch and d) the initial discharge‐charge voltage curves of the Si@CNT/C‐microscroll electrode (for Si‐74, 85, and 92) and for an electrode with 50% Si NPs (SiNP‐50). (a–d) Reproduced with permission.^[^ ^219^ ^]^ Copyright 2020, Royal Society of Chemistry. e) Schematic illustrated for formation procedure, f) SEM images, and g) cycling property f SHCM/NCF composites. (e–g) Reproduced with permission.^[^ ^220^ ^]^ Copyright 2021, Wiley‐VCH.

In **Figure** **21**a, Wang et al.^[^ ^219^ ^]^ developed a flexible, self‐supporting electrode with a high active material content through the self‐rolling of cellulose nanosheets under freeze‐drying conditions. This cellulose‐based topological roll design achieved an unprecedented Si content of 92%, wherein carbon‐coated Si NPs were integrated into 1D microscrolls via CNTs and a carbon cage. In the topological deformation model (Figure 21b), the formation of the microscroll architecture was driven by the warping of the cellulose nanosheets during dehydration and shrinkage, facilitated by the Si NPs. The unique microscroll structure exhibited several advantageous properties: I) the topological microscrolls effectively confined and stabilized the Si NPs within CNT/carbon cages, ensuring uniform dispersion of silicon at high loadings; II) the internal voids within the microscrolls provided ample buffer space to accommodate the volume expansion of silicon during cycling; III) the interconnected microscrolls, supported by CNTs and carbon‐coated Si NPs, maintained good elasticity and electrical conductivity; and IV) the synthesis method for the free‐standing, flexible electrode was simple, green, and cost‐effective. The microscroll electrode membranes, with tunable functionalities, were fabricated through a straightforward compression process (Figure 21c). As a result, the Si@CNT/C‐microscroll anode, containing 85% Si by weight, exhibited excellent cycling stability, delivering 2710 mA h g^−1^ after 300 cycles at 0.2 A g^−1^ and a commercial‐level areal capacity of 5.58 mA h cm^−2^. Notably, at a Si content of 92%, the electrode achieved a high specific capacity of 2704 mA h g^−1^ (Figure 21d).

Additionally, a binder‐free, free‐standing paper electrode was fabricated by interconnecting self‐assembled silicon microspheres and nitrogen‐doped carbon fibers (Figure 21e).^[^ ^220^ ^]^ As shown in Figure 21f, the unique intertwined fabric structure of this electrode effectively suppressed side reactions, facilitated a highly efficient electron/ion conduction network, and alleviated the volume expansion of Si nanodots, resulting in excellent battery performance. The free‐standing paper electrode retained a capacity of 1442 mA h g^−1^ after 800 cycles, with an impressive 86% capacity retention (Figure 21g). When both binder‐free and high mass loading strategies are applied to electrode fabrication, it becomes easier to achieve a higher area capacity for Si‐based anodes without sacrificing specific capacity. However, fabricating thick electrodes using the conventional slurry‐casting technique remains a significant challenge. The critical cracking thickness (CCT) is directly influenced by the viscosity and surface tension of the slurry, which can severely limit the achievable mass loading of electrodes. Low slurry concentrations or high capillary pressure during the drying process may lead to electrode cracking. Overcoming this challenge requires the development of high‐concentration, high‐viscosity conductive binders to enable high mass/area (M/A) or capacity/area (C/A) Si‐based anodes.^[^ ^221^ ^]^ To address this issue, Zhang et al.^[^ ^222^ ^]^ introduced MXene nanosheets as a new conductive binder to fabricate high mass‐loading Si/MXene anodes using a simple and scalable slurry‐casting technique. The MXene inks formed a continuous metallic network around the Si NPs, maintaining the mechanical integrity of the electrode. As a result, the thick electrode (up to 450 µm) achieved an exceptionally high areal capacity of 23.3 mAh cm^−2^.

In summary, advanced self‐healing conductive binders can effectively alleviate the stress caused by the volume expansion of Si anodes during cycling, enhance the formation and stability of the SEI film, and improve the electronic conductivity of the electrode. However, the large size of Si particles reduces the effectiveness of bonding, especially when using self‐healing or gel‐based binders. Additionally, the reversible interactions (e.g., hydrogen bonds) in certain binders may become irreversible after prolonged cycling, leading to a decline in the binder's effectiveness. In contrast, the 3D structure and high mechanical strength of binder‐free electrodes allow them to accommodate a higher proportion of active material (Si) while maintaining the electrode's structural integrity. However, due to limitations in processing technology and production costs, binder‐free electrodes encounter considerable challenges in large‐scale manufacturing, making them more suitable for applications in flexible electronics. To achieve commercial viability, it is crucial to overcome the challenges related to binder‐Si incompatibility, irreversible changes in binder structure, and the difficulties in manufacturing high‐load electrodes.

### 3.4 Silicon‐Based Anode Modified Based on Prelithiation

The construction of a SEI film on the Si surface, coupled with the prior compensation of active Li^+^, offers an effective strategy to mitigate issues associated with SEI film formation during cycling.^[^ ^223^, ^224^, ^225^ ^]^ However, the reduction decomposition potential of the electrolyte component is generally higher than that of the lithium‐silicon alloying potential, preventing the onset of the lithium‐silicon alloying reaction before the electrolyte components undergo reduction and decomposition.^[^ ^226^ ^]^

Prelithiation has emerged as a critical strategy to address these challenges.^[^ ^227^, ^228^, ^229^, ^230^, ^231^ ^]^ Lucht et al.^[^ ^232^ ^]^ recently explored electrochemical prelithiation by varying the distance between the working and counter electrodes. This approach facilitated the formation of a LiF‐rich SEI film (<500 nm) on the Si surface. Electrochemical prelithiation with a battery pretreated at ≈2 mm spacing exhibited the best electrochemical performance. However, this method requires disassembly of the mold and secondary assembly of the full‐cell device after electrode removal, thereby increasing the complexity and cost of manufacturing.^[^ ^228^, ^233^ ^]^

**Figure 22:**
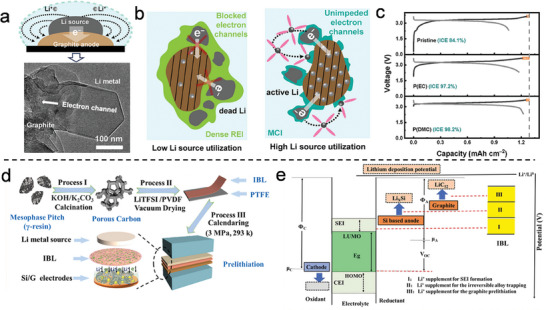
a) Schematic illustration of the contact prelithiation with electron/ion channels and the corresponding TEM image. b) The effect of the dense REI and MCI on the Li source conversion of contact prelithiation. c) Initial charge–discharge curves of the full cells with different lithiated anodes at 0.1 C rate. (a–c) Reproduced with permission.^[^ ^234^ ^]^ Copyright 2022, Wiley‐VCH. d) Schematic illustration of the regulated homogeneous prelithiation process by intermediate buffer layer and e) the corresponding stepwise prelithiation process. (d,e) Reproduced with permission.^[^ ^235^ ^]^ Copyright 2022, Elsevier Ltd.

In contrast, contact prelithiation and chemical prelithiation represent simpler alternatives for constructing the SEI film. Zhang et al.^[^ ^234^ ^]^ developed a novel method utilizing a single‐solvent electrolyte composed of dimethyl carbonate and 1.0 m lithium bis(trifluoromethanesulfonyl)imide (LiTFSI) to modify the structure and composition of the SEI film. This approach slows the closure speed of the electron transport channel, thereby enhancing the prelithiation effect. The transmission electron microscopy image (**Figure** **22**a) clearly shows an electron channel between irregular flakes (graphite) and polygons (Li metal). When the electron channel was obstructed by the dense raw electrolyte interface (REI), the formation of dead Li metal halted the prelithiation process (Figure 22b). In contrast, the DMC‐derived SEI film, acting as a mixed ion/electron conductor interphase (MCI), provided a continuous and smooth electron channel, significantly improving lithium source utilization (92.8%). Full cells assembled using different lithiated anodes with LiFePO_4_ (LFP) cathodes demonstrated that DMC‐based prelithiation resulted in the highest ICE of 98.2% (Figure 22c).

Addressing uneven prelithiation is a critical challenge in the development of silicon‐based anodes. Ma et al.^[^ ^235^ ^]^ implemented a solution to achieve uniform prelithiation on the surface of the Si/G anode by carefully regulating the contact time of the ion/electron pathway and employing a mechanical calendering process (Figure 22d). This method was designed to prevent the loss of active Li^+^ in the full cell by ensuring the formation of a uniform SEI film. The homogeneous prelithiation process for the Si/G anode, which includes an intermediate buffer layer, is illustrated in Figure 22e. In this approach, when the electrochemical potential (µA) of the anode falls below the lowest unoccupied molecular orbital (LUMO) of the carbonate electrolyte, the electrolyte spontaneously undergoes reduction and passivation, forming a stable SEI film. The prelithiation process first compensates for the Li+ loss caused by SEI formation (Region I). As the open‐circuit voltage (OCV) of the cell continues to decrease, the process then compensates for the lithium loss in the high‐potential Si (Region II). Finally, graphite completes Li^+^ intercalation to form LiC_12_ after the deep prelithiation (Region III). By carefully regulating these stages, this approach ensures a more uniform and controlled prelithiation process, which significantly enhances the overall performance of silicon‐based anodes.

**Figure 23:**
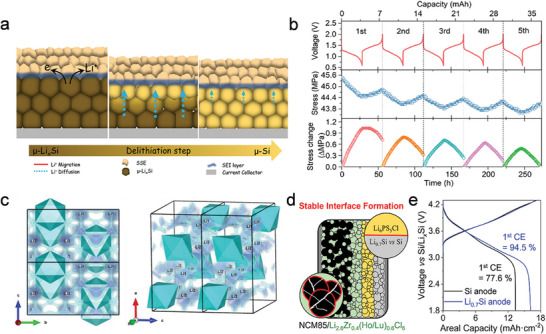
a) Illustration of the delithiation process of an all‐electrochem‐active µ‐Li_x_Si electrode. b) The voltage profiles of µ‐Li_x_Si|SSE|LTO cell (up) along with correlated evolution of stress (middle) and stress change (bottom). (a,b) Reproduced with permission.^[^ ^237^ ^]^ Copyright 2022, Elsevier Ltd. c) The orthorhombic‐II Li_3–x_Zr_x_M_1–x_Cl_6_ obtained using the BVSE method. d) The assembly diagram of ultrahigh‐loading ASSBs with Si anode or Li_0.7_Si anode and e) the corresponding cycling performances. (c–e) Reproduced with permission.^[^ ^238^ ^]^ Copyright 2023, American Chemical Society.

The loss of active Li^+^ is a significant factor contributing to low energy density, making prelithiation a crucial step in designing solid‐state full batteries. In one approach, Si particles were encapsulated with a Li₃PO₄ layer and a carbon shell to form micro‐Si@Li_3_PO_4_@C.^[^ ^236^ ^]^ The Li_3_PO_4_ layer was designed to replenish lithium, resulting in an improved ICE of 83.3%, compared to 74.2% for micro‐Si@Li_3_PO_4_ and 78.3% for micro‐Si@C. In another study, µ‐Li_x_Si alloys were used as an anode material, replacing traditional Si, to create an all‐solid‐state LixSi‐S battery. This system, which featured a 100 wt.% LixSi anode and an argyrodite Li₆PS₅Cl electrolyte, demonstrated excellent cycling stability, with 76% capacity retention over 500 cycles at 0.3C.^[^ ^237^ ^]^ The enhanced cycling capacity was attributed to minimized electrolyte‐related interfacial degradation, as Li_x_Si exhibits relatively soft, highly electronically conductive properties and high lithium diffusivity (**Figure** **23**a). In situ stress evolution of the µ‐LixSi electrode (Figure 23b) showed highly reversible stress changes during charge–discharge cycles, which were linked to the elastic deformation of the SSE layer and the rearrangement of void spaces within the full cell.

Designing electrolytes with high ionic conductivity is another effective strategy to improve electrochemical performance under high area capacity conditions. Nazar et al.^[^ ^238^ ^]^ demonstrated silicon‐based solid‐state batteries using Li_3‐x_Zr_x_M_1‐x_Cl_6_ (0 ≤ *x* ≤ 0.8; M = Ho or Lu) electrolytes (Figure 23c), which exhibited a high ionic conductivity of 1.8 mS cm⁻¹ and a low activation energy of 0.34 eV. The silicon‐based solid‐state batteries were assembled with a Si/prelithiated Li_0.7_Si anode and a high‐nickel Ni LiNi_0.85_Co_0.1_Mn_0.05_O_2_ (NCM85) cathode (Figure 23d). The Li_0.7_Si//NCM85 all‐solid‐state battery achieved a high areal capacity of 16.1 mAh cm⁻^2^, along with a remarkable ICE of 94.49% (Figure 23e).

The prelithiation technology effectively alleviates solid‐liquid interface instability and capacity decay caused by volume expansion through partial lithiation of the Si anode and the pre‐formation of a stable SEI film. However, the SEI film formed during this process often lacks sufficient mechanical strength and Li^+^ conductivity, which can lead to capacity loss in subsequent cycles. To enhance the stability of the SEI film, improvements can be made by optimizing the prelithiation conditions or modifying the surface of the Si anode. These adjustments help control the depth of prelithiation and the formation rate of the SEI film, thereby improving both its uniformity and stability. Additionally, using highly stable electrolytes and additives, such as lithium salts and additives, can further enhance the stability of the SEI film. For large‐scale production, the prelithiation process must also be efficient, cost‐effective, and consistent. Therefore, addressing the compatibility of the prelithiation method with existing production lines and improving the stability of prelithiated materials in humid environments are essential for successful commercialization.

## 4 Summary and Outlook

**Figure 24:**
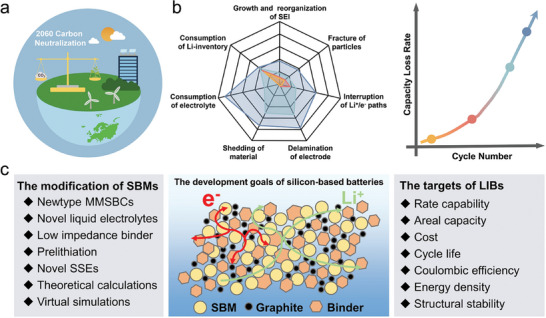
a) Schematic illustration of a balance between carbon emissions and economic development in the future. b) The effects of potential capacity decay factors on the performance of Si‐based full battery during cycling. (b) Reproduced with permission.^[^ ^239^ ^]^ Copyright 2021, Wiley‐VCH. c) Comprehensive insights on how to develop high‐performance silicon‐based full batteries. Reproduced with permission.^[^ ^178^ ^]^ Copyright 2021, Wiley‐VCH.

By 2060, the development of high‐capacity silicon‐based anodes will be essential to overcoming the current bottleneck in the energy density of lithium‐ion batteries, a crucial step toward achieving carbon neutrality (**Figure** **24**a). Silicon‐based anodes are particularly attractive due to their high theoretical specific capacity, low redox potential, and abundant availability of resources. To this end, we have summarized the electrochemical performance data of typical silicon‐based half‐cells and full‐cells in **Table** **1**. However, the commercialization of silicon‐based anodes has been significantly hindered by the drastic volume changes during cycling and sluggish electrochemical kinetics, which are particularly problematic for full batteries. These factors dynamically influence the capacity decay during cycling, as illustrated in Figure 24b.^[^ ^239^ ^]^ To facilitate the industrialization of silicon anodes, it is crucial to address these challenges. This review begins by examining the preparation and modification techniques for MSBMs, analyzing their structural advantages and failure mechanisms, and ultimately proposing strategies to construct high‐energy‐density devices. It offers valuable insights for advancing the comprehensive application of silicon‐based anodes in energy storage systems (Figure 24c). While significant progress has been made in laboratory research, it is important to acknowledge that substantial work remains before these technologies can be fully integrated into industrial‐level applications. Given the interdependence of the internal components in a full battery, several perspectives presented in this review are focused on the modification of MSBMs to improve performance and facilitate practical deployment.

1) New Type of Silicon‐Based Anodes: In energy storage devices, which are often used in limited space, reducing the mass percentage of non‐active components and increasing areal capacity are key strategies to achieve higher energy density in LIBs. To operate effectively under high areal capacity conditions, the electrode must have a rich ion/electron transfer pathway, high electrochemical reactivity, and maintain structural stability at high tap densities. Companies like Betray New Energy Technology Co., Ltd., Shanshan New Energy Technology Development Co., Ltd., Tesla Inc., and Ningde Amperex Technology Co. Ltd., have already achieved 300 Wh kg^−1^ in LIBs. By incorporating Si composites and compensating for Li^+^ in the graphite anode, energy density in silicon‐based full cells can be improved. For instance, in a soft‐pack battery with an NCM811 cathode, silicon‐carbon anode materials with specific capacities of 450, 550, and 800 mAh g^−1^ can increase the full battery energy density to 295, 310, and 330 Wh kg^−1^, respectively. However, as silicon content increases, challenges related to cycle stability and safety become more pronounced. Optimizing silicon‐based anodes can help reduce volume changes and shorten Li^+^ diffusion paths, enhancing lithium storage performance at high tap densities and volume energy densities. Despite the promising performance, the cost of commercial Si/C composites (13.12 CNY kg^−1^) is still twice that of artificial graphite, and the cost of LIBs based on Si/C composites (1.3 CNY Wh^−1^) is double that of conventional graphite‐based batteries. Therefore, developing cost‐effective, mass‐producible, and high‐capacity silicon‐based anodes is critical for improving the energy density of full‐battery devices. In full‐cell applications, fast charge performance is a crucial factor limiting the widespread adoption of LIBs. The low ion/electron transport rates in silicon‐based anodes restrict Li^+^ and electron movement, leading to increased polarization under high current conditions. This issue is exacerbated in low‐temperature environments, negatively impacting the battery's dynamic performance and endurance. Achieving optimal power matching between the anode and cathode is also challenging when the capacity matching between the positive and negative electrodes is not ideal. Thus, developing simple manufacturing processes for high‐power Si/C anode materials is essential to facilitate their commercial application.
2) Novel liquid electrolytes: During cycling, the continuous volume expansion of silicon‐based anodes causes the SEI film on the surface of the active material to repeatedly break, exposing fresh surfaces that consume more electrolytes. Although conventional electrolytes incorporate FEC as a film‐forming agent to enhance SEI film stability, forming a highly mechanically stable SEI film remains challenging. Furthermore, the liquid electrolyte can react with the cathode to form its own SEI film, complicating the system. Therefore, developing a novel electrolyte system that meets the needs of both the anode and cathode is critical. Additionally, advanced in situ or ex situ techniques are needed to explore the relationship between SEI film composition and its mechanical stability, providing guidance for the design of new electrolyte systems. Further investigation of the interactions between the Si surface and various substances, along with an understanding of their dynamic evolution during battery operation, is crucial. Clarifying the surface design principles and electron/Li^+^ transport mechanisms at the interface layer is essential to improve overall battery performance.
3) Low impedance binder: Binders are crucial for maintaining the structural integrity of the electrode. Traditional binders struggle to absorb the internal stresses caused by the repetitive volume expansion of silicon‐based anodes during cycling, leading to the disruption of the 3D conductive network and a decline in electrochemical performance. To improve cycle stability, researchers have focused on developing binders based on non‐covalent bonds (e.g., hydrogen bonds, ionic bonds, host‐guest interactions) or covalent bonds to create self‐healing, 3D binders with strong adhesion. However, binders, as inactive components, tend to reduce the loading of active material within the electrode. Developing low‐impedance binders with high ionic and electronic conductivity could serve a dual purpose: acting both as a binder and as a conductive agent. This would help achieve higher energy densities in LIBs while maintaining structural integrity.
4) Prelithiation: During the initial Li^+^ intercalation, electrolyte decomposition on the surface of MSBMs results in the formation of organic‐inorganic composite films, leading to substantial active Li^+^ loss. This process primarily involves the irreversible release of lithium ions and SEI film formation. For practical applications, the ICE of MSBMs should exceed 95%. To mitigate Li^+^ loss during cycling, MSBMs can undergo prelithiation during material synthesis or electrode preparation. Prelithiation can also pre‐expand the MSBMs, improving the overall cycle performance of the full battery. However, current prelithiation methods (e.g., lithium powder, lithium tape, or electrochemical prelithiation) raise significant safety concerns and involve complex processes. Therefore, developing a safe, efficient, and scalable prelithiation method is essential for commercial applications.
5) Novel SSEs: Although practical applications often include a protective shell, LIBs may still experience short circuits due to external physical shocks, resulting in electrolyte decomposition, swelling, and even thermal runaway. Substituting liquid electrolytes with SSEs is a promising solution to prevent continuous SEI film formation and mitigate volume expansion in MSBMs. However, the complex production process and high cost of SSEs hinder their mass production, making it challenging to integrate them into silicon‐based all‐solid‐state batteries. Therefore, developing efficient processing methods for novel SSEs with low cost, stable interfaces, and high ionic conductivity is crucial for advancing high‐safety, high‐energy‐density LIBs.
6) The combination of theory and experiment: The degradation of electrochemical performance is closely linked to the breakdown of the overall battery structure. Extensive efforts have been made to examine the internal structure and morphology changes of MSBMs, as well as the by‐products formed during cycling, using ex situ or in situ characterization techniques. Theoretical models and virtual simulations have also proven valuable in optimizing performance and understanding specific phenomena. Investigating the capacity contribution of each active component in MSBMs is essential for optimizing electrode formulations. A combination of theoretical calculations and in situ tests can guide LIB material design and full battery development, offering insights into the interactions between the anode, cathode, and electrolyte. Furthermore, theoretical simulations can be employed for pre‐experimentation or extreme environment testing, providing cost savings and shortened production cycles. Thus, the integration of theory and experiment is a powerful approach to designing low‐cost, mass‐produced, high‐energy‐density LIBs.
7) The mechanical and electrochemical coupling: The investigation of silicon‐based anodes aims not only to improve electrochemical performance but also to address the mechanical stresses induced by volume changes during lithium‐ion transport. In liquid‐state batteries, the electrolyte can mitigate the impact of stress changes on the electrode‐electrolyte interface. However, in solid‐state batteries, stress‐induced mechanical damage to the interface between the electrode and electrolyte is both irreversible and significant. Researchers are increasingly recognizing the importance of mechanical‐electrochemical coupling. We believe that future applications of MSBMs must involve a comprehensive exploration of the interplay between mechanical stresses and electrochemical behavior to ensure long‐term performance and stability.

**Table 1:** Recent progress on Si‐based anode for half‐cells and full cells.

Half‐cells					Full‐cells			
Anode	ICE	Cycling performance	Loading mass	Rate performance	Cathode	Cycling performance	Energy density	Reference
onion‐like Si/C	84.5%	1391 mAh g^−1^ at 0.2 A g^−1^ after 400 cycles	0.8 mg cm^−2^	63.9% capacity retention at 2 A g^−1^	/	/	/	Energy Storage Mater. 24 (2020) 312–318.
Si/G@C/TiN	/	776.5 mAh g^−1^ at 5 A g^−1^ after 400 cycles	0.8 mg cm^−2^	660 mAh g^−1^ at 10 A g^−1^	NMC523	133.7 mAh g^−1^ at 165 mA g^−1^ after 50 cycles	≈476 Wh kg^−1^ based on the total weight of active materials	Energy Storage Mater. 29 ((2020) 367–376.
	81%	1050 mAh g^−1^ at 0.84 A g^−1^ after 1000 cycles	0.8 mg cm^−2^	873.7 mAh g^−1^ at 10 A g^−1^	/	/	/	Adv. Funct. Mater. 30 (2020) 1910249.
subnano‐sized Si composite	93.1%	1262.3 mAh g^−1^ at 0.5 C after 50 cycles	7.5 mg cm^−2^	/	NCM811	91.24% capacity retention after 2875 cycles	/	Nat. Energy. 6 (2021) 1164–1175.
Pitch‐derived carbon/Si nanolayer/graphite composite	90.9%	/	7 mg cm^−2^	65% capacity retention at 5 C	90% of NCM and 10% of NCA	81.9% capacity retention after 200 cycles	/	Adv. Energy Mater. 9 (2019) 1803121.
Si‐layers in the graphite macropores composites	93%	493.9 mAh cm^−3^ after 100 cycles	6.9 mg cm^−2^	/	LiCoO_2_	1825.7 Wh L^−1^ after 100 cycles	2363.6 Wh L^−1^	Nat. Commun. 10 (2019) 475.
Si MPs@graphene networks	82.6%	774 mAh g^−1^ at 1.0 A g^−1^ after 1000 cycles	2.3 mg cm^−2^	/	NMC811	1.8 mAh cm^−2^ at 1.0 mA cm^−2^ after 100 cycles	1048 Wh L^−1^	Natl. Sci. Rev. 8 ((2021) nwab12.
Si composites with high graphite carbon shell covalent bonding	/	750 mAh g^−1^ at 2.0 A g^−1^ after 500 cycles	3.05 mg cm^−2^	790 mAh g^−1^ at 5.0 A g^−1^	NMC811	2.94 mAh cm^−2^ after 100 cycles at 95 mA g^−1^	/	Adv. Energy Mater. 13 (2023) 2300874.
Graphene‐coated disproportionated SiO	78.2%	72.4% capacity retention at 2.0 A g^−1^ after 500 cycles	/	774.0 mAh g^−1^ at 5.0 A g^−1^	LiFePO_4_	75% capacity retention after 100 cycles	/	Adv. Funct. Mater. 31 (32) (2021).
SiOx@TiO_2_@C	81.2%	89.5% capacity retention (vs. 2nd discharge) after 800 cycles	/	949.7 mAh g 1 at 10 A g 1	C‐NMC811	2 mAh cm^−2^ at 2 A g^−1^ after 100 cycles		Nano Energy 77 (2020) 105082.
Carbon‐coated ant‐nest‐like microscale porous Si	80.3%	1271 mAh g^−1^ at 2.1 A g^−1^ after 1000 cycles	0.8 mg cm^−2^	1712 mA h cm^−3^ at 420 mA g^−1^	NMC111	84% capacity retention after 400 cycles	502 Wh Kg^−1^	Nat. Commun. 10 (2019) 1447.
Core‐shell gradient porous Si material	76.5%	1059 mAh g^−1^ even after 500 cycles at 2 A g^−1^	/	1916 mAh g^−1^ at 4 A g^−1^	LiCoO^2^	89.6% capacity retention at 70 mA g^−1^ after 50 cycles	/	Adv. Funct. Mater. 32 (2022) 2107897.
Mesoporous Si@Si@G	88.7%	1246 mAh g^−1^ at 2.1 A g^−1^ after 300 cycles	0.68 mg cm^−2^	/	LiCoO_2_	1.75 mAh cm^−2^ at 0.8 mA cm^−2^ after 100 cycles	/	Nano Energy. 61 (2019) 404–410.
Gt‐SiNW	72%	80% capacity retention of (vs. 10th cycle capacity) at C/5 after 200 cycles	2.7 mg cm^−2^	327 mAh g^−1^ at 5 C	NMC622	70% capacity retention after 300 cycles	414 Wh kg^−1^	ACS Nano. 14 (2020) 12006–12015.

NMC111 is Li(Ni_1/3_Co_1/3_Mn_1/3_)O_2_, NMC622 is LiNi_0.6_Co_0.2_Mn_0.6_O_2_, NMC523 is LiNi_0.5_Co_0.2_Mn_0.3_O_2_, and NMC811 is LiNi_0.8_Co_0.1_Mn_0.1_O_2_.

In the development of high‐performance silicon‐based full batteries, it is essential to consider practical aspects from the perspective of enterprises and large‐scale applications. Currently, several laboratory‐scale methods show excellent performance, but they face significant challenges when translated to large‐scale production (**Table** **2**). These challenges include high production costs, complex processing conditions, and the use of expensive raw materials. On the other hand, methods that are scalable and cost‐effective may struggle with issues such as material performance, quality consistency, and long‐term stability. Therefore, addressing the unique challenges of each silicon‐carbon material preparation method is critical. Achieving a balance between scalability, cost‐effectiveness, and product performance is one of the major hurdles facing the industry. Enterprises must carefully assess these methods, considering specific production requirements, material needs, and budget constraints.

**Table 2:** Shortcomings of common silicon‐carbon material preparation methods: A practical perspective.

	Technical disadvantages	Enterprise and application challenges
Ball milling (mechanical alloying)	Inhomogeneous distribution of silicon and carbon Mechanical degradation of silicon particles	High energy consumption Poor reproducibility and inconsistent product quality Material waste powder
Chemical vapor deposition	High temperatures Insufficient carbon layer uniformity and adhesion.	High equipment and material costs Complex process control Material degradation and yield reduction at high temperatures
Sol‐gel process	Low conductivity Poor mechanical properties	Low yield and long production time Increased energy consumption and operating costs due to secondary high‐temperature treatments: Low material purity:
Hydrothermal synthesis	Poor structural integrity High temperature and pressure requirements	High energy consumption Difficulties in scaling up
Direct carbonization of precursor materials	Incomplete carbonization Poor control of carbon content:	Poor material consistency due to variability in precursor quality Extended production cycle due to long carbonization time

In summary, overcoming the primary challenges of silicon‐based anodes—such as achieving high capacity, stability, and cost‐effectiveness—requires an integrated approach. This approach must combine innovative materials, advanced electrolytes, efficient manufacturing processes, and a deeper understanding of mechanoelectrochemical kinetics. Striking the right balance between these factors is crucial for meeting the growing demands of the energy storage market while optimizing the production process. The future success of silicon‐based anodes hinges on overcoming these obstacles and translating promising laboratory‐level innovations into commercially viable, high‐performance energy storage solutions.

## Conflict of Interest

The authors declare no conflict of interest.
